# Expanded roles and divergent regulation of FAMA in Brachypodium and Arabidopsis stomatal development

**DOI:** 10.1093/plcell/koac341

**Published:** 2022-11-28

**Authors:** Katelyn H McKown, M Ximena Anleu Gil, Andrea Mair, Shou-Ling Xu, Michael T Raissig, Dominique C Bergmann

**Affiliations:** Department of Genetics, Stanford School of Medicine, Stanford, California 94305, USA; Biology Department, Stanford University, 371 Jane Stanford Way, Stanford, California 94305, USA; Biology Department, Stanford University, 371 Jane Stanford Way, Stanford, California 94305, USA; Howard Hughes Medical Institute, Stanford University, 371 Jane Stanford Way, Stanford, California 94305, USA; Department of Plant Biology, Carnegie Institution for Science, 260 Panama St., Stanford, California 94305, USA; Biology Department, Stanford University, 371 Jane Stanford Way, Stanford, California 94305, USA; Biology Department, Stanford University, 371 Jane Stanford Way, Stanford, California 94305, USA; Howard Hughes Medical Institute, Stanford University, 371 Jane Stanford Way, Stanford, California 94305, USA

## Abstract

Stomata, cellular valves found on the surfaces of aerial plant tissues, present a paradigm for studying cell fate and patterning in plants. A highly conserved core set of related basic helix-loop-helix (bHLH) transcription factors regulates stomatal development across diverse species. We characterized BdFAMA in the temperate grass *Brachypodium distachyon* and found this late-acting transcription factor was necessary and sufficient for specifying stomatal guard cell fate, and unexpectedly, could also induce the recruitment of subsidiary cells in the absence of its paralogue, BdMUTE. The overlap in function is paralleled by an overlap in expression pattern and by unique regulatory relationships between BdMUTE and BdFAMA. To better appreciate the relationships among the Brachypodium stomatal bHLHs, we used in vivo proteomics in developing leaves and found evidence for multiple shared interaction partners. We reexamined the roles of these genes in *Arabidopsis thaliana* by testing genetic sufficiency within and across species, and found that while BdFAMA and AtFAMA can rescue stomatal production in Arabidopsis *fama* and *mute* mutants, only AtFAMA can specify *Brassica*-specific myrosin idioblasts. Taken together, our findings refine the current models of stomatal bHLH function and regulatory feedback among paralogues within grasses as well as across the monocot/dicot divide.

## Teaser

Comparative expression and functional analysis of the stomatal differentiation gene FAMA in Brachypodium and Arabidopsis reveals variation in cell fate acquisition and maintenance.

IN A NUTSHELL

**Background:** Plants are essential players in global carbon and water cycling. The structures central to this role are stomata—pores on leaf surfaces. Plants open and close their stomata depending on the environmental conditions to regulate gas exchange, and can adjust the number of stomata they make. Tuning stomatal numbers and activity can improve water use efficiency and drought tolerance. Typically, two guard cells flank a stomatal pore, but grasses have additional subsidiary cells flanking each guard cell, making their stomata even better at responding to environmental cues. What genes are involved in creating such magnificent stomata?

**Question:** We knew that homologs of three stomatal genes first found in Arabidopsis—*SPCH*, *MUTE*, and *FAMA*—were in other plants, and that SPCH and MUTE could take on unexpected roles across species. The sequence of FAMA from *Brachypodium distachyon*, a relative of wheat, differs in intriguing ways from Arabidopis FAMA, so we asked what roles *BdFAMA* plays in Brachypodium stomatal development.

**Findings:** *BdFAMA* promotes guard cell fate in the final step of stomatal development, but unexpectedly, is “on” much earlier, overlapping with the preceding gene, *BdMUTE*. Other grasses die without *MUTE* activity, but in Brachypodium, this earlier expression of *BdFAMA* can compensate for the absence of *MUTE*. In Arabidopsis, the BdFAMA function can replace both *MUTE* and *FAMA*, but cannot rescue myrosin cells, a specialized cell type in the Brassica family. So, some of *FAMA's* roles are the same across different plants with unique stomata, but other roles have species-specific nuances.

**Next steps:** To improve crops, we need to understand how plant cells and organs are made and specialized. This work in the temperate grass Brachypodium provides a framework that future scientists can use to connect environmental signals to stomatal development and regulation and contribute to improving agriculturally significant grasses like wheat and barley.

## Introduction

**Figure 1:**
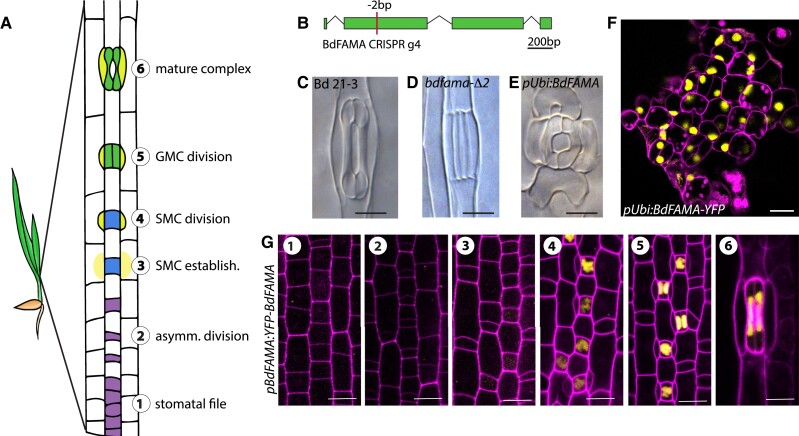
BdFAMA is required for stomatal guard cell differentiation. A, Diagram of stomatal development in the developmental zone of a *Brachypodium distachyon* leaf, with Stages 1–6 as follows: A stomatal file is established (Stage 1; purple) and, following an asymmetric division (Stage 2), the smaller daughter cell gives rise to a guard mother cell (GMC; blue). GMCs induce subsidiary mother cell (SMC) formation from laterally adjacent cells (Stage 3), which divide asymmetrically to produce subsidiary cells from the smaller daughter cell (SCs; Stage 4; yellow). GMCs divide symmetrically to form guard cell (GC) precursors (Stage 5; green), which differentiate to form a mature stomatal complex (Stage 6), with paired GCs and SCs flanking a pore. B, Gene diagram of *BdFAMA* with the location of CRISPR/Cas9-generated 2-bp deletion. C–E, Differential interference contrast (DIC) microscopy images of stomata from cleared leaf tissue in WT (Bd21-3), (C) *bdfama-*Δ*2* (D), and *Ubi_pro_:BdFAMA* (E). F, Confocal image of ectopic, unpaired guard cells induced by *Ubi_pro_:BdFAMA-YFP* (yellow). G, Confocal images of *BdFAMA_pro_:YFP-BdFAMA* reporter expression during stomatal development. No *YFP-BdFAMA* expression is detected during stomatal file establishment and asymmetric division (Stages 1 and 2). *YFP-BdFAMA* (yellow) is detected at low levels in GMCs during SMC establishment (Stage 3) and increases in expression during SC recruitment (Stage 4). *YFP-BdFAMA* peaks in newly formed GCs (Stage 5) and persists in mature stomatal complexes (Stage 6). DIC images taken of cleared abaxial epidermal tissue: images of *bdfama-*Δ*2* were from the second leaf of 11 days post-germination (dpg) T4 plants, *Ubi_pro_:BdFAMA* and *Ubi_pro_:BdFAMA*-YFP are from T0 regenerants. Confocal images from the abaxial surface of second leaf 6 dpg plants, cell walls (magenta) stained with propidium iodide (PI). Scale bars: 10 μm. See also Supplemental Figure 1.

Cell fate establishment is essential for creating cellular diversity, tissue development, and organ formation—effectively, all aspects of organismal development. Stomatal development represents a paradigm for studying cell fate acquisition following asymmetric cell divisions. Stomata, present in nearly all land plants, are cellular valves found on the surfaces of aerial plant tissues which regulate carbon dioxide and water vapor exchange. Stomata are typically made up of two kidney-shaped guard cells (GCs) flanking a pore, but the shape and patterning of these structures on a leaf can be clade specific. The kidney-shaped GCs of dicots like Arabidopsis (*Arabidopsis thaliana*) are distributed in a pattern that follows a one-cell spacing rule, where little order exists beyond the prohibition of stomata in direct contact (Yang and Sack, 1995; Geisler *et al*., 2000). This pattern emerges from asymmetric and oriented divisions of dispersed stem cell-like precursors, called meristemoids, in the young leaf epidermis. Stomata in grasses have a distinctly different morphology, featuring dumbbell-shaped GCs flanked by a pair of subsidiary cells (SCs) derived from adjacent, lineally unrelated, cell files. Their ontogeny is also highly ordered, and the grass stomatal lineages develop sequentially from the base of a developing leaf in epidermal cell files adjacent to those overlying veins (Figure 1A).

Arabidopsis *FAMA* (*AtFAMA*) was the first transcriptional regulator published as being essential for stomatal fate (Bergmann *et al*., 2004; Ohashi-Ito and Bergmann, 2006). Multiple studies have since linked this factor to both the acquisition and the continued maintenance of the differentiated state, the latter through direct interaction with the cell cycle regulator RETINOBLASTOMA RELATED (RBR; Hachez *et al*., 2011; Lee *et al*., 2014; Matos *et al*., 2014). *AtFAMA* has additional roles in cell cycle control; in *atfama* mutants, epidermal tumors form by the repeated symmetric cell divisions of incompletely differentiated GCs (Ohashi-Ito and Bergmann, 2006). *AtFAMA* and its two closest paralogues, *SPEECHLESS* (*AtSPCH*) and *AtMUTE*, are basic helix-loop-helix (bHLH) transcription factors in the group Ia subfamily (Pires and Dolan, 2010), which initiate the asymmetric divisions of the stomatal lineage and ensure commitment to GC fate, respectively (Ohashi-Ito and Bergmann, 2006; MacAlister *et al*., 2007; Pillitteri *et al*., 2007). Expression of *AtFAMA* produces single cells with hallmarks of stomatal GC identity in the absence of *AtSPCH* and *AtMUTE*. However, hyperactivation of these earlier-acting TFs cannot overcome the requirement for *AtFAMA* to produce GCs (Ohashi-Ito and Bergmann, 2006). AtFAMA is also required as part of a negative regulatory loop with AtMUTE to ensure that stomata consist of exactly one pair of sister GCs (Han *et al*., 2018). To bind DNA and effect transcriptional change, AtSPCH, AtMUTE, and AtFAMA require heterodimerization with functionally redundant group IIIb bHLH partners INDUCER OF CBF EXPRESSION1/SCREAM (AtICE1/AtSCRM) and AtSCRM2 (Kanaoka *et al*., 2008).

The genetic origin and duplication history of SPCH, MUTE, FAMA, ICE1/SCRM, and SCRM2 have been tracked among extant land plants, and there is evidence for deep conservation and connection to stomatal identity (Chater *et al*., 2016). Among flowering plants, putative orthologues of *AtFAMA* have been readily identified (Macalister and Bergmann, 2011; Chater *et al*., 2016; Raissig *et al*., 2016; Ortega *et al*., 2019; Wu *et al*., 2019). Preliminary functional analysis of mutants in rice (*Oryza sativa*) indicates that *OsFAMA* is necessary for the production of functional GCs, though not for cell cycle inhibition, as *osfama* mutants arrest with two incompletely differentiated GCs and SCs (Liu *et al*., 2009; Wu *et al*., 2019). Moreover, other bHLHs have undergone clade-specific gene duplications. For example, compared with Arabidopsis, the temperate grass model Brachypodium (*Brachypodium distachyon*, Pooidae) has an additional *SPCH* and these two *BdSPCH* paralogs have partially redundant roles in lineage initiation. Both Arabidopsis and Brachypodium have two SCRM-like genes, but they are derived from independent duplications and exhibit different degrees of functional redundancy in stomatal lineage activities (Kanaoka *et al*., 2008; Raissig *et al*., 2016). Recent data have also shed light on functional divergences within the grasses. The single *MUTE* gene, which acquired a new role in SC recruitment in grasses, is essential in maize (*Zea mays*) and rice, but not in Brachypodium (Raissig *et al*., 2017; Wang *et al*., 2019; Wu *et al*., 2019). The differential requirement for *MUTE* to produce viable stomata suggests that specialization can occur even within this well-conserved gene family, and such specialization may extend to other elements of the stomatal lineage developmental regulatory network.

Of the key players in stomatal development, *SPCH1* and *SPCH2*, *MUTE*, *SCRM2*, and *ICE1* have all been characterized in Brachypodium (Raissig *et al*., 2016, 2017). Multiple phylogenetic analyses position Bradi2g22810 as the putative Brachypodium *FAMA* homolog (Wu *et al*., 2019; Buckley *et al*., 2020; McKown and Bergmann, 2020), but expression and function of this final “stomatal bHLH” have not yet been reported. Evidence from phylogenetic and sequence homology support *FAMA* as being closest to an ancestral form from which the group Ia paralogs were derived (Macalister and Bergmann, 2011). Unlike SPCH and MUTE, the function of FAMA in GC differentiation is completely dependent on an intact DNA binding domain (Davies and Bergmann, 2014). Although the GC differentiation role of *FAMA* is conserved across land plants, FAMA has demonstrated additional functions in Arabidopsis, such as cell division control and enforcement of terminal cell fate through chromatin remodeling, and it has been co-opted into the development of a *Brassica*-specific non-epidermal cell type (Shirakawa *et al*., 2014). However, whether the roles of FAMA extend beyond its GC differentiation capacity in other plant systems is not known. In this study, we characterize the endogenous roles of *BdFAMA* and find that it is necessary and sufficient for specifying stomatal GC fate. Our data show that *BdFAMA* expression commences much earlier in the stomatal lineage than expected, given its terminal role in GC differentiation, and this extended expression may allow *BdFAMA* to compensate for the absence of *BdMUTE* in a way that is distinct from the other grasses. Because the loss of *BdFAMA* resembles the loss of *BdSCRM2*, and BdFAMA and BdMUTE have overlapping functions, we characterized the diversity of bHLH complexes through in vivo proteomics in developing Brachypodium leaves and found evidence for multiple shared interaction partners. The overlaps in *BdFAMA* and *BdMUTE* function then prompted us to reexamine the roles of these paralogues in Arabidopsis. By testing genetic sufficiency within and across species, we found that *BdFAMA* and *AtFAMA* can rescue stomatal production in Arabidopsis *fama* and *mute* mutants, but that only *AtFAMA* can participate in the specification of *Brassica*-specific myrosin idioblasts. Taken together, our findings prompted us to reconsider the current models of stomatal bHLH function and enabled us to refine our understanding of regulatory feedbacks among paralogues.

## Results

### Functional analysis of *BdFAMA*

To characterize the role of the putative Brachypodium *FAMA* homolog (BdFAMA, Bradi2g22810) in stomatal development, we used clustered regularly interspaced short palindromic repeats (CRISPR)-CRISPR-associated nuclease 9 (Cas9)-mediated genome editing in the wild-type line, Bd21-3, to induce mutations early in the second exon of the coding region. As expected from the essential nature of *FAMA* in other species, T0 regenerants harboring homozygous frameshift mutations were seedling lethal. We obtained a transgenic plant that is heteroallelic for a six-base pair (bp) and a 2-bp deletion, with the latter predicted to result in a frameshift and a premature stop codon in the second exon, prior to the bHLH domain (Figure 1B; Supplemental Figure 1, A–C). Among offspring of this line, plants homozygous for *bdfama-*Δ*2* completely lack functional stomata, and instead, exhibit a four-celled complex of undifferentiated, paired GCs with flanking SCs; Figure 1, C and D). The same defective stomatal phenotype is observed in T0 regenerants bearing other homozygous frameshift mutations (Supplemental Figure 1D). In contrast to the *fama* “tumors” found in Arabidopsis, there were no continued guard mother cell (GMC) divisions in *bdfama*, which supports a role for *BdFAMA* in GC differentiation, but not necessarily cell division control. The undifferentiated four-cell complex phenotype is strikingly similar to the phenotype resulting from a loss of BdFAMA's predicted partner, *BdSCRM2*, but differs from that seen from the loss of *BdICE1*, the other potential heterodimer partner (Raissig *et al*., 2016).

Loss of *BdFAMA* resulted in a discrete phenotype of GC differentiation failure without disturbing general leaf shape and pattern. However, a broad overexpression of *BdFAMA* (*Ubi_pro_:BdFAMA-YFP* and *Ubi_pro_:BdFAMA*) results in severely deformed leaves where most leaf epidermal cells are converted into kidney-shaped cells that accumulate localized cell wall material resembling a pore (Figure 1, E and F; Supplemental Figure 1E). The vast majority of these cells were unpaired and oriented such that their pore-like region faced the leaf tip, a phenotype that resembles the overexpression of *AtFAMA* in Arabidopsis (Ohashi-Ito and Bergmann, 2006). Occasionally, paired GCs with flanking cells resembling SCs are seen near files adjacent to cells overlying veins (Figure 1E), which may suggest that cells already fated to become GCs retain some normal patterning, whereas non-stomatal cells transdifferentiate when exposed to BdFAMA. Taken together, these results suggest *BdFAMA* is necessary and sufficient to promote GC fate.

To monitor *BdFAMA* expression in developing leaves, we created transcriptional (*BdFAMA_pro_:3xYFP*) and translational (*BdFAMA_pro_:YFP-BdFAMA*) reporters. We anticipated that *BdFAMA* would be restricted to the final stage of stomatal development, consistent with the loss of terminal GC differentiation in *bdfama. BdFAMA_pro_:YFP-BdFAMA,* however, could already be detected weakly in young GMCs during the timeframe of subsidiary mother cell (SMC) establishment. This reporter peaks before the GC division and persists into mature GCs (Figure 1G). A similar pattern is seen with the transcriptional reporter (Supplemental Figure 1F). The *BdFAMA* expression window overlaps considerably with that of *BdMUTE* (Raissig *et al*., 2017) during SC recruitment, GC division, and early GC differentiation; although unlike *BdMUTE*, *BdFAMA* does not appear to be expressed in SMCs or SCs. *BdFAMA*'s temporally broad and continued expression contrasts with that in Arabidopsis, where *AtFAMA* peaks in young GCs, and both translational reporters (Adrian *et al*., 2015; Han *et al*., 2018) and single-cell RNA sequencing (scRNA-seq) data (Lopez-Anido *et al*., 2021) show *AtMUTE* and *AtFAMA* expression initiate sequentially, and that they share little overlap in protein expression. The difference in bHLH expression windows between Arabidopsis and Brachypodium suggests there are alternative regulatory strategies at play that have yet to be discovered.

### *BdMUTE* is involved in regulating *BdFAMA* during development

**Figure 2:**
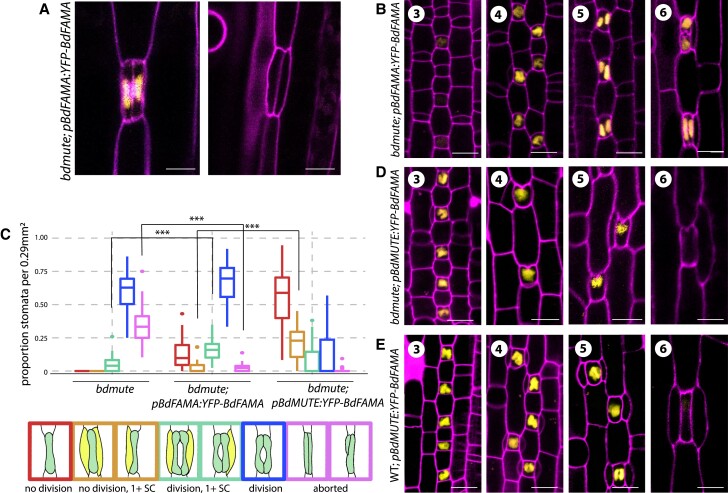
BdFAMA recruits subsidiary cells in the absence of *BdMUTE*. A–B, Confocal images of abaxial tissue of *BdFAMA_pro_:YFP-BdFAMA* in stomatal complexes of *bdmute; BdFAMA_pro_:YFP-BdFAMA*. A, *YFP-BdFAMA* is present in differentiated complexes but is absent in aborted GCs. B, Confocal images of stomatal development in *bdmute; BdFAMA_pro_:YFP-BdFAMA* during SMC establishment (Stage 3) and SC recruitment (Stage 4), in newly formed GCs (Stage 5) and mature stomatal complexes (Stage 6). C, Quantification of stomatal phenotypes in *bdmute* and *bdmute* expressing either *BdFAMA_pro_:YFP-BdFAMA* or *BdMUTE_pro_:YFP-BdFAMA* from cleared abaxial tissue of the second leaf, 6–7 days post germination (dpg). Cartoon representations of phenotypic classes are shown at the bottom, outlined in the colors represented in the plot. For each sample, five different regions of the leaf were imaged and quantified. *n* = 6 leaves, each from individual plants, for *bdmute* and *bdmute; BdFAMA_pro_:YFP-BdFAMA*, and *n* = 8 for *bdmute; BdMUTE_pro_:YFP-BdFAMA*. ***P < 0.001 (based on a Wilcoxon rank sum test, followed by Bonferroni correction for multiple comparisons). In each boxplot, the colored horizontal line indicates the median, upper, and lower edges of the box (hinges) representing the upper and lower quartiles, and whiskers extend to the largest observation within 1.5 interquartile ranges of the box. D, E, Confocal images of stomatal development in abaxial tissue of *bdmute; BdMUTE_pro_:YFP-BdFAMA* and of wild-type; *BdMUTE_pro_:YFP-BdFAMA* T0 regenerants at indicated stages. Cell outlines (magenta) visualized by propidum iodide (PI) staining. Scale bars: 10 μm. See also Supplemental Figures 2 and 3.

In *bdmute*, the predominant phenotype is failure to recruit SCs, such that stomatal complexes consist solely of a pore flanked by two GCs with kidney-shaped rather than dumbbell-shaped morphologies (Raissig *et al*., 2017). In addition, 40% of the complexes also show defects in GC differentiation, and instead produce long, paired cells that resemble the undifferentiated GCs of the *bdfama* mutant. In the mature leaf zone of *bdmute*, these paired cells never take on GC fate, and instead, appear to leave the stomatal lineage, elongating in a way similar to pavement cells. Based on these phenotypes, we hypothesized that *BdMUTE* is involved in the expression or regulation of *BdFAMA*, and that the aborted GCs were a result of insufficient BdFAMA activity. To test regulatory relationships between *BdMUTE* and *BdFAMA*, we created transgenic lines expressing the *BdFAMA* translational reporter in a *bdmute* background (Figure 2, A and B). We detected YFP-BdFAMA in all mature, differentiated GCs in *bdmute; BdFAMA_pro_:YFP-BdFAMA*. However, we did not observe YFP signal in the undifferentiated complexes, which suggests that their failure to differentiate was indeed due to the lack of *BdFAMA*. It is unclear whether the absence of *YFP-BdFAMA* in aborted GCs was due to a complete failure to activate *BdFAMA* expression in stomatal precursors or a failure to maintain sufficient levels of BdFAMA. Nevertheless, the data suggest that *BdMUTE* likely plays a role in the stabilization of *BdFAMA* expression during development.

### *BdFAMA* recruits subsidiary cells in *bdmute*

In surveying phenotypes in the multiple independent transformed lines of *bdmute; BdFAMA_pro_:YFP-BdFAMA*, we noticed that many also exhibited a significant reduction in the occurrence of aborted GCs. Using T4 plants from a representative line (line 19; all subsequent references to *bdmute; BdFAMA_pro_:YFP-BdFAMA* phenotypes will refer to this line), we quantified this phenotype and found that aborted GCs are reduced from ∼34% in *bdmute* to ∼2% in *bdmute; BdFAMA_pro_:YFP-BdFAMA* (Figure 2C, aborted). A new phenotype emerged where some GMCs completely failed to divide symmetrically, but the single *YFP-BdFAMA*-positive GMCs acquired a dumbbell shape, as if their GC differentiation program is intact; however, these single GCs did not form a pore (Figure 2C, no division).

A second unexpected phenotype of *bdmute; BdFAMA_pro_:YFP-BdFAMA* was the successful recruitment of SCs to stomatal complexes. About 19% of complexes, including those whose GMCs did or did not divide symmetrically, are able to recruit one or both SCs (Figure 2C, 1+ SC categories). The presence of SCs suggests *BdFAMA* can partially compensate for *BdMUTE*. This ability of *BdFAMA* to compensate for the loss of *BdMUTE* is especially interesting, given that the cell–cell mobility of BdMUTE was hypothesized to enable it to recruit SCs from cell files neighboring the GMC (Raissig *et al*., 2017). Our *BdFAMA* translational and transcriptional reporters are detected exclusively in GMCs in both *bdmute* and wild-type Bd21-3 plants, so there is no evidence that the BdFAMA protein can move between cells (Figures 1G and 2B; Supplemental Figure 1F). While many of the complexes with successful SC recruitment had typical SCs—appropriately proportioned to the length and size of its stomatal complex—many SCs are abnormally large and arise from diagonal SMC divisions, and it is unclear whether these large SCs would be able to work in concert with the GCs to facilitate rapid pore opening and closing (Supplemental Figure 2A). Many of the GMC divisions were also abnormal and produced two asymmetric GC precursors (rather than two symmetric cells) or oriented their division planes perpendicular to the normal placement. Misorientation of GMC division occurs in both *bdmute* and *bdmute; BdFAMA_pro_:YFP-BdFAMA*, so there does seem to be some requirement of *BdMUTE* for proper placement of the division plane that is not improved with additional BdFAMA (Supplemental Figure 2B). Despite BdFAMA's inability to improve the placement of the GMC division plane, these data show that the non-mobile YFP-BdFAMA is capable of driving SC recruitment in the absence of BdMUTE, even when expressed under the BdFAMA promoter.

### Earlier expression of *BdFAMA* causes GMC division failure

While partial complementation of *bdmute* was possible with BdFAMA expressed under its own promoter, successful SC recruitment seemed to occur later in development than in the wild-type. To test whether rescue of SC recruitment in *bdmute* improved with an earlier expression of BdFAMA, we created *BdMUTE_pro_:YFP-BdFAMA* in the *bdmute* background. Most T0 regenerants died as young plantlets, and in tissue imaged from these individuals (*n* = 13 severe lines, *n* = 6 milder lines), we observed that GMCs fail to divide symmetrically to produce paired GCs and instead produce single GCs with a dimple resembling a pore (pseudo-pores), oriented toward the leaf tip (Figure 2D). These pseudo-pores also began forming in GMCs during the SC recruitment stage, indicative of premature BdFAMA activity (Figure 2D, Stage 3). Approximately 21% of these single GC complexes are able to recruit one or more SC (Figure 2C). In some lines, single GC complexes are stacked end-to-end, violating the one-cell spacing rule, and a large pore with underlying airspace is formed that allows a stunted, pale green plant to persist and produce a few T1 seeds, which enabled us to quantify the relative expression levels across the lines (Supplemental Figure 3, A and B). We observed a dose-dependent effect on GC division and plant growth; individuals in which GCs fail to divide symmetrically and cause lethality or stunted plant growth express more *YFP-BdFAMA* than lines with infrequent GC division phenotypes that have normal growth (Supplemental Figure 3, C–E).

We hypothesized that the GMC division failure was caused by the premature activity of *BdFAMA* only in the absence of *BdMUTE*, and that normally BdMUTE acts as a temporal placeholder that, through competition for binding partners or target promoter binding sites, ensures that differentiation does not commence until SCs are recruited and the GMCs have divided. To test this hypothesis, we expressed *BdFAMA* driven by the *BdMUTE* promoter (*BdMUTE_pro_:YFP-BdFAMA*) in a wild-type background. We observed a dose-dependent effect: many T0 regenerants produced stomatal complexes with single GCs like in *bdmute; BdMUTE_pro_:YFP-BdFAMA* (*n* = 7 severe lines). However, more lines (*n* = 10 mild lines) had an intermediate or milder phenotype, where only some of their GCs failed to divide symmetrically. Additionally, if single GCs generate ectopic leaf-tip facing pores, they appear only after SC recruitment (Figure 2E). These data support a role for *BdMUTE* in the proper timing of *BdFAMA* activity.

### BdMUTE and BdFAMA have shared and distinct interaction partners

**Figure 3:**
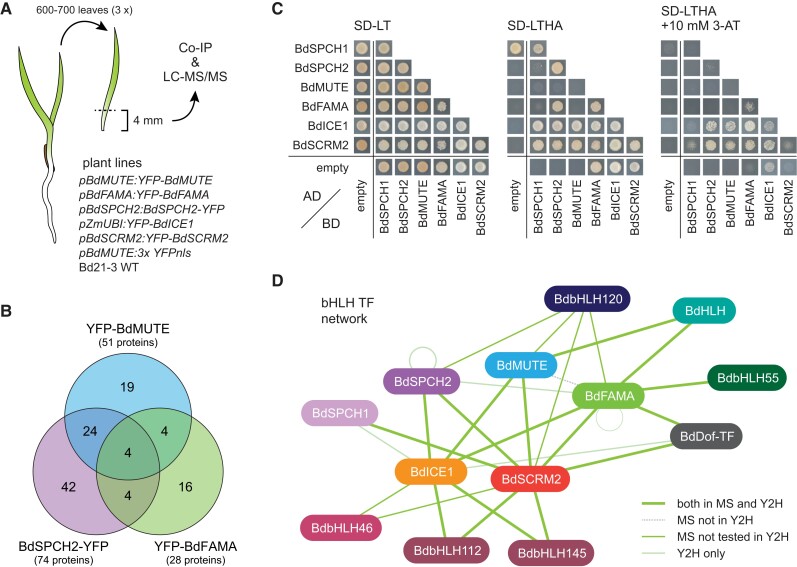
Identification of physical interaction partners of Brachypodium stomatal bHLHs. A, Cartoon diagram of sampling strategy and plant lines used to identify bHLH interactors in vivo. The developmental zones (bottom 4 mm) of 600–700 leaves per sample (three replicates of each of the seven genotypes listed) were harvested and used for co-immunoprecipitation (co-IP) with GFP-Trap beads. Bound proteins were identified by LC-MS/MS. B, Venn diagram showing the overlap of specific putative interaction partners found for BdMUTE, BdFAMA, and BdSPCH2. C, Yeast two-hybrid (Y2H) assay testing interactions between the six master regulators of stomatal development. Yeast transfected with the indicated activation domain (Ad) and binding domain (BD) fusion proteins was spotted on a synthetic defined medium without leucine and tryptophane (SD-LT) or leucine, tryptophane, histidine, and alanine (SD-LTHA) to test for successful transfection and protein interaction, respectively. 3-AT was added to increase stringency of the selection and overcome auto-activation. D, Stomatal bHLH TF network. Line styles indicate whether an interaction was identified in the co-IP experiment (MS) and/or in the Y2H assays. See also Supplemental Figures 4–8.

How do *BdMUTE* and *BdFAMA* maintain their functional specificity with such overlap in expression and function? Based on their similar mutant phenotypes, one might speculate that there is a preference for BdFAMA/BdSCRM2 heterodimers and BdSPCHs/BdICE1 heterodimers (Raissig *et al*., 2016). However, whether BdICE1 or BdSCRM2 preferentially heterodimerize with *BdMUTE* is not known. To better understand the functional overlap between BdMUTE and BdFAMA, we investigated their in vivo binding partners using co-immunoprecipitation (co-IP) and subsequent analysis with liquid chromatography coupled with tandem mass spectrometry (LC-MS/MS). We sampled tissue from the proximal 4 mm of leaves, a region that encompasses the peak expression of stomatal development genes, from lines stably transformed with YFP-tagged BdSPCH2, BdMUTE, BdFAMA, and BdSCRM2 expressed under their native promoters, and BdICE1 expressed under a maize *Ubiquitin* promoter (Figure 3A; Supplemental Figures 4 and 5). A *BdMUTE* promoter-driven nuclear-targeted triple YFP-tagged line and wild-type Bd21-3 were used as controls.

With our model of preferred heterodimer pairing, BdFAMA/BdSCRM2 and BdSPCHs/BdICE1, we would expect to find these specific partner pairings from our pull-down interaction data. However, the LC-MS/MS data show that all assayed group Ia bHLHs, BdSPCH2, BdMUTE, and BdFAMA, can associate in vivo with both group IIIb bHLHs, BdICE1 and BdSCRM2. Despite possibly having shared heterodimer binding partners, we found that both BdMUTE and BdFAMA pulled down overlapping as well as unique interaction candidates. Of the candidate interactors identified, only eight are shared between BdFAMA and BdMUTE, which, based on our pipeline for defining enriched interactions, pull down 28 and 51 total interactors, respectively (Figure 3B; Supplemental Figures 6 and 7; Supplemental Data Set 1). Notably, the number of shared interactors between BdMUTE and BdFAMA is comparable with their overlap with BdSPCH2, suggesting that shared interactors alone cannot explain the functional overlap of *BdFAMA* with *BdMUTE*. BdMUTE also pulled down BdFAMA, which suggested that the two might even exist in a shared complex. We also identified several transcription factors among interacting proteins, mainly bHLHs, that could act as alternative dimerization partners or contribute to a higher order bHLH complex.

We confirmed these interactions in vitro with a yeast two-hybrid (Y2H) assay and found that BdSPCH2, BdMUTE, and BdFAMA are able to interact with both BdICE1 and SCRM2 (Figure 3C; Supplemental Figure 8A). We could not confirm the interaction between BdMUTE and BdFAMA, and it is possible that these associate through a higher order complex rather than a direct interaction (Figure 3C). Interaction of other transcription factors identified in the co-IP experiment could also be confirmed by Y2H (Supplemental Figure 8B). A helix-loop-helix (HLH) transcription factor that might act as a transcriptional inhibitor interacted with both BdMUTE and BdFAMA; bHLH55, for which an Arabidopsis ortholog was found to interact with AtFAMA (Mair *et al*., 2019), interacted with BdFAMA; bHLH112 and bHLH145, which are orthologous to AtbHLH71, interacted with BdICE1 and BdSCRM2; and a Dof TF that showed similarity to STOMATAL CARPENTER (SCAP1), a GC morphology regulator in Arabidopsis (Negi *et al*., 2013), interacted with BdICE1 and BdSCRM2, and weakly with BdFAMA. The summary of core bHLH interaction data in Figure 3D shows that although there is apparent flexibility of heterodimer pairings possible between bHLHs, unique interactions remain prevalent. Additionally, while there still might be preferred heterodimer binding partners that could influence the functional specificity of the core stomatal bHLHs, it is not on a level that we were able to detect from these approaches. However, the possibility remains that BdFAMA could achieve the functional flexibility needed to substitute for *BdMUTE* by timing- or dosage-dependent alternate binding with BdICE1 or BdSCRM2.

### *BdFAMA* drives the GMC to GC cell fate transition in the absence of *BdMUTE*

In contrast to Brachypodium, maize and rice plants do not survive the loss of *MUTE* activity (Wang *et al*., 2019; Wu *et al*., 2019). GMCs in these cereal crops fail to recruit SCs and divide longitudinally to form GCs, arresting instead as small cells of unclear identity after several misoriented divisions (Wang *et al*., 2019; Wu *et al*., 2019). Our observations that an extra copy of *BdFAMA* can compensate for *BdMUTE* in SC recruitment suggested that, in Brachypodium, endogenous *BdFAMA* is also poised to drive GMC to GC fate transitions when *BdMUTE* is missing. If this hypothesis were correct, then a double mutant of *bdmute; bdfama* would resemble the single *mute* mutants of maize and rice.

**Figure 4:**
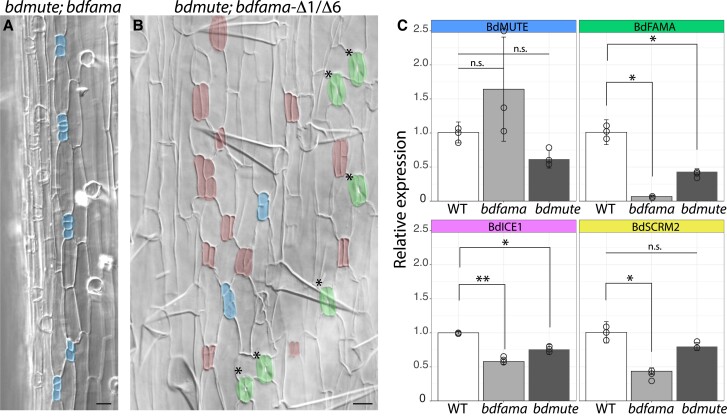
Genetic evidence for differential redundancy of *MUTE* and *FAMA* among the grasses. A, B, DIC images of cleared abaxial tissue of *bdmute; bdfama* T0 regenerants. A, Severe *bdmute; bdfama* regenerant with no correctly specified guard cells (GCs) and groups of small cells (blue false color) resembling the small arrested cell phenotype in rice and maize *mute* mutants. B, Milder phenotype of sequence-confirmed heteroallelic *bdmute*; *bdfama-*Δ*1/*Δ*6* with mature stomata lacking subsidiary cells (green false color), aborted GCs (red false color), and arrested small cells (blue false color). C, Relative expression from reverse transcription quantitative PCR (RT-qPCR) of stomatal genes from tissue collected in the leaf division zone of wild-type, *bdfama*, and *bdmute* plants. Expression values were normalized to control gene *BdUBC18* and are relative to expression in wild-type plants. Data presented are means ± standard deviation. n.s. = Not significant. Asterisks indicate significant differences compared with wild-type expression (Welch's *t*-test followed by Bonferroni multiple comparison correction); **P < 0.01, *P < 0.05. See also Supplemental Figure 9.

We created double *bdmute; bdfama* mutants using the same *BdFAMA* CRISPR guide and Cas9 vector that we previously used to generate *bdfama*. The regenerants (28 total from two independent transformations) were very sick and rarely grew larger than a few millimeters in length before dying and, in many cases, this precluded our ability to collect enough tissue to both image and genotype individuals. While all T0 regenerants generated were lethal, there were two classes of mutant severity. Similar to maize *bzu2/zmmute*, the severe class of regenerants fail to produce any stomata or recruit SCs, and instead, small-celled GMCs arrest after a few rounds of division (Figure 4A). Less severe mutants are able to produce a handful of SC-less stomatal complexes, complete with a pore, but most complexes arrest as small cells or paired, undifferentiated, aborted GCs, like those found in *bdmute* (Figure 4B). We were able to genotype a regenerant from the less severe group, and it was heteroallelic for 1 and 6 bp deletions in the second exon of *BdFAMA* (*bdmute*; *bdfama-*Δ1/Δ6). The increased number of aborted GCs in mild *bdmute; bdfama* lines and the complete absence of them in the severe lines suggested that aborted GCs in *bdmute* had, at one time, expressed BdFAMA, but upon longitudinal division, lost expression and were unable to complete differentiation. These data show that lacking even one functional copy of BdFAMA in the bdmute background can cause lethality and mimic the phenotype of *bzu2/zmmute* and *osmute*, and is evidence that BdFAMA is indeed able to drive the cell fate transition from GMC to GC in the absence of *BdMUTE*.

### Regulatory relationships within stomatal bHLHs have diverged within the grasses

The genetic and transcriptional data from Arabidopsis support a model in which AtMUTE is required for *AtFAMA* expression (Pillitteri *et al*., 2007; Han *et al*., 2018). In maize and rice, there is almost a complete reduction of *FAMA* expression in *mute* backgrounds (Wang *et al*., 2019; Wu *et al*., 2019), suggesting that, like in Arabidopsis, there is *MUTE-*dependent initiation of *FAMA* expression. However, our observations of the BdFAMA reporters and phenotypes suggest that the regulatory strategy in Brachypodium may be less *MUTE* dependent. To further define the relationships between *BdMUTE* and *BdFAMA* and their implications for the stomatal lineage, we used reverse transcription quantitative PCR (RT-qPCR) to measure expression levels of stomatal bHLHs in *bdmute, bdfama*-Δ2, and wild-type (Figure 4C). While *BdFAMA* expression was reduced in the *bdmute* background compared with the wild-type, we still detected considerable amounts of transcript. Reduced expression may also be a consequence of the 40% of stomatal complexes that failed to differentiate (Raissig *et al*., 2017) and lacked BdFAMA reporter expression in *bdmute*. Thus, BdMUTE may be involved in the stabilization of BdFAMA RNA or protein accumulation, but it is not essential for the initiation of *BdFAMA* expression. The *bdmute* allele we used in this study (*bdmute-1/sid*) has a 5-bp deletion at position 523–527 bp in the first exon, and our primers amplify the region between 157 and 219 bp; thus, the detection of *BdMUTE* expression in the *bdmute* background is likely from a partial transcript. The *bdmute-1* allele has been considered a complete loss of function, however, because its mutant phenotype is indistinguishable from CRISPR/Cas9-generated null lines (Raissig *et al*., 2017). *BdSCRM2* and *BdICE1* expression levels were lower in both *bdmute* and *bdfama* backgrounds, but the reduction was more pronounced in the *bdfama* background, which likely corresponds to the reduction of stomatal lineage cells due to the failure of stomatal complex maturation. Remarkably, *BdMUTE* expression increased in *bdfama* compared with the wild-type. Together, these data support a model wherein reciprocal regulation between BdFAMA and BdMUTE is required for proper developmental progression, where BdMUTE promotes the expression of *BdFAMA*, and BdFAMA restrains *BdMUTE*. Although *BdFAMA* levels were reduced in *bdmute*, our ability to still detect *BdFAMA* points to a MUTE-independent activation of *BdFAMA* as a reason for BdFAMA's compensatory abilities, while the MUTE-dependent activation of FAMA results in lethality in Arabidopsis, rice, and maize *mute* mutants.

### *BdFAMA* rescues GC differentiation but incompletely enforces terminal fate in Arabidopsis

**Figure 5:**
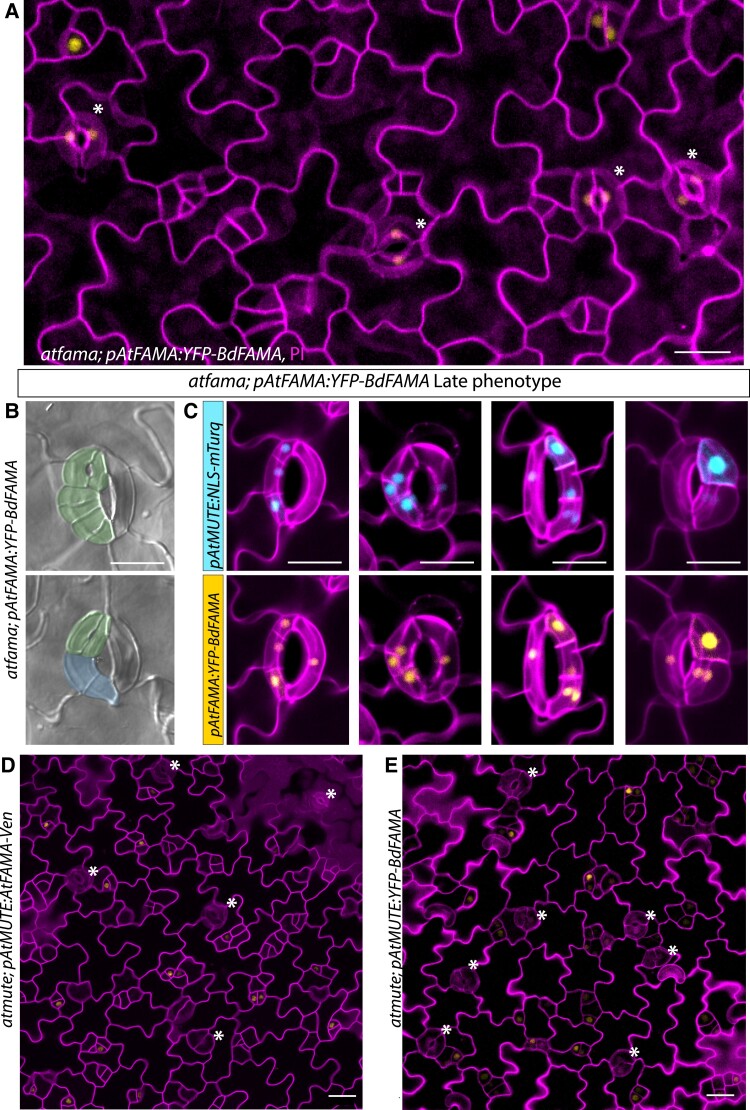
BdFAMA rescues Arabidopsis guard cell production but is unable to fully enforce terminal fate. A, Confocal image of *atfama; AtFAMA_pro_:YFP-BdFAMA* at 5 days post germination (dpg) with white asterisks indicating mature stomatal complexes. B, DIC images of abaxial cleared tissue from *atfama; AtFAMA_pro_:YFP-BdFAMA* at 16 dpg. Pseudocolor denotes guard cells (GCs; green) and guard mother cells (GMCs; blue). C, Confocal images of reprogrammed guard cells forming stomata within stomata in late stage (12–16 dpg) abaxial cotyledons from *atfama; AtFAMA_pro_:YFP-BdFAMA; AtMUTE_pro_:NLS-mTurq*. mTurq (blue) and YFP (yellow) channels are shown individually with cell wall staining (magenta). D and E, Confocal images of *atmute; AtMUTE_pro_:AtFAMA-Ven* and *atmute; AtMUTE_pro_:YFP-BdFAMA* from 5 dpg abaxial cotyledons. D, *AtFAMA* can rescue *atmute*. E, *BdFAMA* can rescue *atmute*. White asterisks indicate stomata. Cell wall outlines (magenta) were visualized by propidium iodide (PI) staining. Scale bar: 20 μm. See also Supplemental Figure 10.

FAMA promotes GC differentiation in rice, maize, and Brachypodium, but it appears that even within the grasses, the regulation of *BdFAMA* and *BdMUTE* has evolved in distinct ways. Across the monocot/dicot divide, key domains of FAMA are well conserved (Supplemental Figure 9), and FAMA is similarly required for GC differentiation, but it is additionally required for cell cycle control and terminal fate enforcement in Arabidopsis. Given this difference, we asked whether BdFAMA can fully or partially rescue cell fate and cell cycle defects in *atfama*. To ascertain the degree of rescue, we approached phenotypic analysis with the following criteria: to what extent is BdFAMA able to (1) induce GC fate, (2) prevent excessive GMC divisions, and (3) enforce terminal GC fate? We created lines expressing *AtFAMA_pro_:YFP-BdFAMA* in a heterozygous *atfama*/+ background, and genotyping of individual T2 plants indicated that we could obtain living *atfama* homozygous plants, suggesting that BdFAMA can rescue the GC fate defect in *atfama*. These *AtFAMA_pro_:YFP-BdFAMA; atfama* plants have numerous normal-looking stomata, and overall, resemble wild-type Arabidopsis in stomatal density and patterning (Figure 5A).

We did observe occasional clusters of small epidermal cells in the rescued lines, but they did not resemble the over-proliferating GC “tumors” typical of *atfama* mutants (Ohashi-Ito and Bergmann, 2006). To get a more detailed picture of the capacity of BdFAMA to substitute for AtFAMA, we introduced an *AtMUTE* transcriptional reporter (*AtMUTE_pro_:NLS-mTurq*) into *atfama; AtFAMA_pro_:YFP-BdFAMA* (Supplemental Figure 10A). A time course analysis at 3, 4, and 5 days post germination (dpg) reveals that the small cells in these clusters arise from cells that express low levels of *AtMUTE* (Supplemental Figure 10A, 4 dpg), but not all of these cells later express BdFAMA and give rise to stomata; instead, *AtMUTE*-expressing cells divide again and daughter cells often express different levels of *AtMUTE* (Supplemental Figure 10A, 5 dpg). Expression of *AtMUTE* demarcates GMC fate and commitment to the stomatal lineage (Pillitteri *et al*., 2007), so the continued division of *AtMUTE*-expressing cells could be caused by the imperfect ability of BdFAMA to drive the GMC fate transition in Arabidopsis, but does not necessarily imply a defect in cell division enforcement.

AtFAMA interacts with the Arabidopsis homolog of the cell cycle inhibitor RETINOBLASTOMA RELATED (RBR) through an LxCxE binding motif (Matos *et al*., 2014; Supplemental Figure 9), and this interaction confers terminal GC fate through chromatin modifications (Lee *et al*., 2019). Without this FAMA–RBR interaction, GCs lose their identity and can begin to lobe like pavement cells or divide asymmetrically and reinitiate the stomatal lineage to produce a stomata-in-stomata phenotype (Matos *et al*., 2014). Grass FAMAs have an IxCxE motif (Supplemental Figure 9), which may result in a less stable or more transient interaction with RBR (Mcgivern *et al*., 2005). To test whether BdFAMA is sufficient to enforce terminal GC fate, we examined the stomata in the epidermis of older cotyledons (14–17 dpg) in *atfama; AtFAMA_pro_:YFP-BdFAMA* lines and found stomatal complexes that failed to maintain terminal differentiation (Figure 5B). The BdFAMA-induced phenotype resembled the stomata-in-stomata phenotype produced when the AtFAMA LxCxE motif is disrupted, but there are some differences. Notably, GC lobing is less prominent and a single reprogrammed GC could produce several adjacent stomata (Figure 5B). To better characterize the loss of terminal GC fate, we examined the *AtMUTE* transcriptional reporter in *atfama; AtFAMA_pro_:YFP-BdFAMA* (Figure 5C). In stomata whose GCs underwent several divisions, all daughter cells expressed both *AtMUTE_pro_:NLS-mTurq* and *YFP-BdFAMA*; thus, it appeared that rather than reprogramming to the earliest developmental state with the potential to become a pavement cell or commit to stomatal fate, these daughter cells were only reprogrammed to GMC fate. Newly formed GCs expressing YFP-BdFAMA could also be found immediately adjacent to presumed GMCs, indicating a problem with maintaining one-cell spacing. While BdFAMA can rescue stomatal formation and prevent *fama* tumors, the reprogramming of stomata points to the inability of BdFAMA to properly regulate the enforcement of terminal GC fate.

The discovery that *BdFAMA* could substitute for *BdMUTE* contrasted with older work in Arabidopsis that suggested these two proteins had non-overlapping functions based on the failure to identify viable plants when *AtMUTE* and *AtFAMA* were swapped (Macalister and Bergmann, 2011). *Atfama* mutants could, however, be partially rescued by the expression of a *Physcomitrium patens* gene that was considered the progenitor of *SPCH*, *MUTE,* and *FAMA* (Macalister and Bergmann, 2011). To test whether *BdFAMA* could rescue *atmute*, we generated *atmute; AtMUTE_pro_:YFP-BdFAMA*, and used *atmute; AtMUTE_pro_:AtFAMA-Venus* as a specificity control. We frequently observed well-formed stomata in both rescue lines, and both lines produced healthy, viable plants (Figure 5, D and E). This suggests that *AtMUTE* and *AtFAMA* have a greater degree of functional overlap than previously appreciated, despite sharing little sequence similarity aside from the conserved bHLH domain and SPCH-MUTE-FAMA (SMF)/ACT-like domain (Supplemental Figure 9). In both rescue lines, there are adjacent small cells that express YFP-BdFAMA, and at 10 dpg, clusters of single GCs and stomata could be seen (Supplemental Figure 10, B and C). Both lines produced single GCs, which is indicative of premature transdifferentiation to GC fate. These results demonstrate that, despite millions of years of divergence, *BdFAMA* can substitute for *AtFAMA* in most GC functions, but also revealed that the compensatory ability of *FAMA* for *MUTE* is shared in Arabidopsis and Brachypodium.

### *BdFAMA* is unable to induce the formation of *Brassica*-specific myrosin idioblast cells

**Figure 6:**
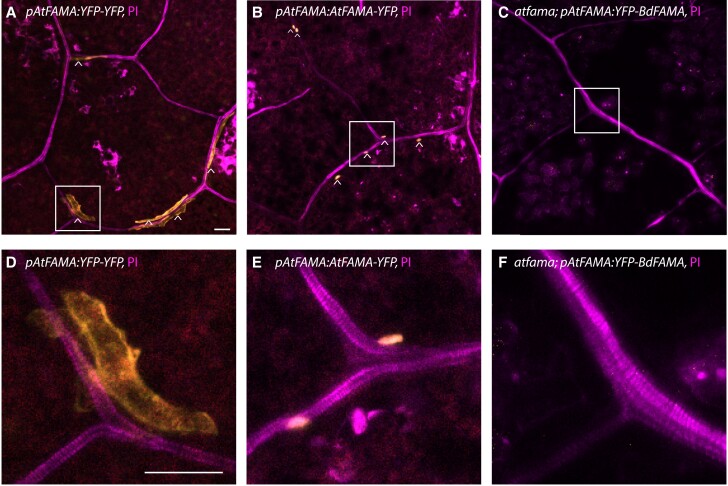
BdFAMA does not rescue myrosin cell differentiation in *atfama*. A, Developing myrosin idioblasts (MIs, white carats) expressing the transcriptional marker *AtFAMA_pro_:YFP-YFP* (yellow). B, Developing MIs (white carats) showing nuclear expression of the translational marker *AtFAMA_pro_:AtFAMA-YFP*. C, *atfama; AtFAMA_pro_:YFP-BdFAMA* fails to rescue MI development and lacks expression of *YFP-BdFAMA*. D–F, Zoomed in images of the white boxes in (A–C), respectively. All confocal images were taken from the first rosette true leaves 10–15 dpg with propidium iodide (PI) cell wall stain. Scale bar: 20 μm. See also Supplemental Figure 10.

The ability of BdFAMA to substitute for AtFAMA in GC fate may not be entirely surprising; GCs are a highly conserved cell type, so it follows that many of the genes required for GC fate and function are also conserved. But does this functional conservation extend if a transcription factor is co-opted into a new role? Arabidopsis, like other *Brassicas*, is armed with a glucosinolate-myrosinase mustard oil defense to protect against microbes and insects (Shirakawa and Hara-Nishimura, 2018). Both GCs and myrosin idioblasts (MIs) accumulate myrosinases for this defense system, and previous works have shown that *AtFAMA* is required for specifying MI fate (Li and Sack, 2014; Shirakawa *et al*., 2014). This cell type is not produced in Brachypodium (nor grasses in general), so we were uniquely positioned to ask whether BdFAMA's ability to rescue *atfama* also extended to this phenotype. MI cells and their nuclei can easily be visualized by the expression of a cytoplasmic *AtFAMA* transcriptional reporter (Figure 6, A and D), and by a functional *AtFAMA* translational reporter (Figure 6, B and E), respectively. Although we could readily identify signals in MIs of the control reporter lines and when *AtFAMA_pro_:BdFAMA*-*YFP* is expressed in a wild-type or *atfama*/+ background (Supplemental Figure 10F), we are unable to detect signal when *AtFAMA_pro_:BdFAMA*-*YFP* is in an *atfama* homozygous background, suggesting that BdFAMA is incapable of rescuing MI fate (Figure 6, C and F). We also visualized mature MI cells using Coomassie brilliant blue (CBB) staining and are able to see the characteristic horned shape of mature myrosin cells in the control line, but have failed to identify them in the *atfama; AtFAMA_pro_:BdFAMA*-*YFP* lines (Supplemental Figure 10, D and E). This demonstrates that BdFAMA is not able to rescue the formation of *Brassica*-specific MI cells.

## Discussion

In this study, we found that *BdFAMA* is necessary and sufficient for specifying GC fate in Brachypodium. Loss-of-function mutants fail to differentiate their GCs, form undifferentiated four-celled complexes, and die as a result. Much of what we already knew about FAMA's role in stomatal differentiation from work in rice and Arabidopsis held true in Brachypodium, but distinct differences emerged by extending our characterization to encompass FAMA's interplay with its paralog MUTE in these species.

We have phenotypic points of comparison in rice with *osfama-1*, but a lack of extensive analysis of transcriptional and translational reporters, along with the inconsistency of *fama* phenotypes between rice studies (Liu *et al*., 2009; Wu *et al*., 2019), have precluded the ability to dig deeper into interesting questions; namely, how does a group of well-conserved transcription factors coordinate to produce the patterning and morphology so unique to the grasses? From our expression analysis, we found that *BdFAMA*'s temporal window begins earlier than expected, given its terminal role and what is known from Arabidopsis, but because grasses lack a comparable self-renewing meristemoid stage, the timing is still consistent with expression initiation in GMCs. However, BdFAMA's maintained expression in mature complexes remains puzzling. Does it serve a functional purpose? Is this the case in other grasses? The model in Arabidopsis is that the AtFAMA/RBR complex ensures terminal GC fate through the deposition of repressive chromatin modifications on stomatal lineage genes such as *SPCH* and *MUTE* (Matos *et al*., 2014). Perhaps, continued production of BdFAMA in mature stomatal complexes is the strategy grasses use to maintain terminal cell fate, rather than through the repressive chromatin modifications mediated through RBR.

### Multiple regulatory strategies coordinate stomatal bHLH activity

*BdFAMA* is not the only bHLH in Brachypodium with a puzzlingly long expression window for its presumed function. *bdscrm2* has a late-arrest phenotype that phenocopies that of *bdfama* and suggests they are heterodimer binding partners, but it is expressed throughout the stomatal lineage. In addition, *bdice1* has an early-stage arrest phenotype similar to the *bdspch1; bdspch2* double mutant, suggesting *BdICE1* forms a heterodimer with *BdSPCHs* (Raissig *et al*., 2016). This supports a strategy where specific heterodimer pairs perform distinct functions, and preferential binding may act as a mechanism for maintaining functional specificity in a lineage with spatiotemporal overlap. This strategy for achieving many functional outcomes from a core set of bHLH TFs has been seen across development, from maize anthocyanin biosynthesis to *Drosophila* neuronal development (Powell and Jarman, 2008; Hao *et al.,* 2021), and recent evidence from Arabidopsis hints that the ACT-like domain present in stomatal bHLHs may contribute to binding selectivity (Seo *et al.,* 2022). However, binding specificity alone cannot explain how functional specificity is maintained, as the interaction data from our co-IP–LC-MS/MS assay show that all combinations between stomatal group Ia and group IIIb bHLHs are possible in vivo.

In a TF family with shared expression timing, heterodimer partners, domains, and the need to coordinate between regulatory and cooperative roles, an additional layer of regulation becomes important. We show that lacking even one functional copy of *BdFAMA* in the *bdmute* background can cause lethality and mimic the phenotype of *bzu2/zmmute* and *osmute*, which is evidence that *BdFAMA* is indeed able to drive the cell fate transition from GMC to GC in the absence of *BdMUTE* in a dosage-dependent manner. We also observed that severe *bdmute; bdfama* mutant GMCs failed to divide longitudinally to specify GCs. Therefore, the aborted GCs seen in the *bdmute* mutant likely expressed BdFAMA earlier in development to drive the GC division but lost expression upon dividing, providing further evidence of dosage dependence, where late expression of BdFAMA is sensitive to BdFAMA dosage and may require stabilization by BdMUTE. Additionally, while *BdSCRM2* has a late-stage role, it is still able to partially complement *bdice1* to initiate stomatal formation when broadly overexpressed (Raissig *et al*., 2016). Together, these data suggest gene dosage acts as another important layer of regulation in grass stomatal development.

### SMC priming can occur without apparent BdFAMA cell–cell mobility

Our genetic comparisons gave us insight not only into the interplay of key bHLHs of the stomatal lineage, but into the recruitment of grass SCs. The current model for SC recruitment posits that after the production of MUTE in GMCs, the TF moves to the neighboring cell file to induce asymmetric division of the SMCs. The demonstrated rescue of *bdmute*'s SC recruitment defect by BdFAMA, however, suggests protein mobility is not an absolute requirement to induce SMC fate. BdFAMA is not mobile, as we did not detect it in any neighboring cell files, so there may instead be mobile intermediaries that BdFAMA is able to activate to prime SMC fate. However, in *bdmute; BdFAMA_pro_:YFP-BdFAMA*, many stomatal complexes failed to properly generate a controlled asymmetric SMC division, and instead, created abnormal SCs or lobed neighboring cells. This may be due to the inability of *BdFAMA* to substitute for a subset of BdMUTE-specific partners or reflect binding site specificity, or may suggest that movement of the TF to the neighboring cell file is required for consistently oriented polarization of the SMC.

These data reveal a distinction between the ability to induce SMC fate and orient the SMC division. Recent work in Brachypodium and maize identified polarity factors involved in SMC division that support this distinction between fate and division control (Cartwright *et al*., 2009; Wang *et al*., 2019; Zhang *et al*., 2022). In both Brachypodium and maize, *Pangloss1* (*PAN1*) polarizes to the SMC/GMC interface and coordinates the nuclear migration and asymmetric SMC division; without *PAN1*, abnormal SCs and incomplete recruitments occur (Cartwright *et al*., 2009; Zhang *et al*., 2022). An additional polarity protein, *BdPOLAR*, was found opposite to the SMC/GMC domain of *BdPAN1* in Brachypodium, and had an additive effect on SMC division orientation control with *BdPAN1* (Zhang *et al*., 2022). BdPOLAR's expression in the SMC is dependent on *BdMUTE*, while the expression and polarization of BdPAN1 still occur in the absence of *BdMUTE*, which explains why we saw some successful SC recruitment in *bdmute* lines expressing YFP-BdFAMA. This is also evidence that these two polarity factors have differential dependence on the priming of SMC identity. In the context of our study, BdFAMA can compensate for priming SMC identity in the absence of BdMUTE if expressed at the right time, but BdMUTE or its mobility is likely needed for consistent, proper polarization of the SMC asymmetric division.

### BdFAMA is functionally conserved for stomatal roles

We have shown that BdFAMA's compensatory abilities run deeper than initially thought, going so far as to prevent the lethality of *bdmute* that is present in all other characterized plants, including grasses. We provide evidence that the regulatory relationship between FAMA and MUTE has diverged in Brachypodium, such that *BdFAMA* is expressed independently of *BdMUTE* and is able to drive the GMC to GC fate transition, but the possibility remains that there are protein domain differences in BdFAMA that aid in its compensation. In addition to the *how,* the *why* of BdFAMA's extracurricular role in the GMC to GC fate transition and SC recruitment is intriguing, and presents an opportunity to explore whether this regulatory divergence emerged from domestication events or phylogeny-driven speciation through comparisons of translational and transcriptional reporters in maize and rice. In analyzing the mutant phenotypes of *MUTE* and *FAMA*, it also became apparent that clearer comparisons are needed for the developmental stages in grasses and Arabidopsis. Grasses do not have a self-renewing meristemoid cell type, but based on the *mute* mutant phenotype in maize and rice, and the *bdmute; bdfama* double mutant in Brachypodium, there does seem to be a pre-GMC stage that requires either *MUTE* or *BdFAMA* to advance. Thus, grass “GMCs” may transition through several distinct developmental stages that may be comparable with Arabidopsis development, but inconsistent nomenclature across species obfuscates direct phenotypic comparisons.

This research shows that even one of the most conserved bHLH family members has specialized roles and regulations in its evolution to produce grass stomata. Grasses do not have the same requirement for dynamic cell cycle control in the stomatal lineage as in Arabidopsis, and *BdFAMA* seems to have greater stability in mature stomatal complexes. However, despite millions of years of divergence, *BdFAMA* seems to have retained its ability to control the cell cycle, even though the Brachypodium loss-of-function phenotype suggests it is not a required role for the lineage. With BdFAMA expression occurring so early in the lineage, it stands to reason that having a strong cell cycle inhibitory role may prove detrimental if stochastic levels of BdFAMA can cause aberrant GMC division failure and reduce stomatal numbers. The grass leaf is also set up with a much stronger developmental zoning than Arabidopsis, and the grass stomatal lineage does not have dividing stem cell-like populations that demand high regulatory plasticity—so cell division control along the leaf may defer to a more global regulatory signal.

MI cells are another example of functional divergence between Arabidopsis (*Brassica*) and grasses. In these cells, the role of FAMA is different; in Arabidopsis, FAMA is expressed in small ground meristem cells and is required, along with AtICE1 or AtSCRM2, for myrosin cell development (Li and Sack, 2014; Shirakawa *et al*., 2016). The requirement of these three bHLHs for myrosin cell differentiation indicates the presence of a shared transcription factor network between myrosin cells and GCs, and yet, *BdFAMA* was unable to rescue the formation of myrosin cells and we failed to detect expression in the absence of AtFAMA. GC vacuoles in the Brassicales also contain myrosinases, so it is thought that *FAMA* was first co-opted into this defense system in stomata, and later, during evolution, acquired a role in the development of myrosin cells (Shirakawa and Hara-Nishimura, 2018). Myrosin cells represent a synapomorphy in *Brassicas*, and although BdFAMA is unable to participate in their formation, the fact that we observed the expression of *AtFAMA_pro_:YFP-BdFAMA* in wild-type and *atfama/+* lines hints at a myrosin-specific regulatory strategy. Specifically, the expression of *AtFAMA* in the Arabidopsis stomatal lineage is MUTE dependent and FAMA independent (Ohashi-Ito and Bergmann, 2006; Han *et al*., 2018). However, *AtMUTE* expression is not detected in myrosin cells (Li and Sack, 2014), so there is a difference in the regulatory relationship between MUTE and FAMA within the Arabidopsis epidermis and inner leaf tissue. As we failed to detect the expression of *YFP-BdFAMA* in the absence of *AtFAMA* but could see the expression in wild-type and *atfama/+* backgrounds, our data suggest that in myrosin cells, AtFAMA is required for its own expression, which is consistent with observations from Li and Sack (2014) and Shirakawa *et al*. (2014). Domain swaps between *AtFAMA* and *BdFAMA* will help to uncover structural features responsible for their differential capabilities, and comparative transcriptomics of *atfama* mutants rescued by AtFAMA or BdFAMA may be useful to determine which genes are required to produce MIs.

This study demonstrated the functional conservation of *BdFAMA* in driving GC fate across the monocot/dicot divide. Importantly, the differences seen in *mute* phenotypes and differential regulation of *FAMA* across the grasses bring to light that our ability to understand the role of one gene hinged on understanding the system as a whole. We showed that with a thorough investigation of transcriptional and translational markers, in addition to leveraging genetic manipulations within and across species, it is possible to get a more complete picture of the players in pathways such as the stomatal lineage. Furthermore, the existence of reporter lines for each bHLH transcription factor in the Brachypodium stomatal lineage enabled our pursuit of in vivo proteomics that produced interaction data useful for identifying candidates involved in stomatal development and regulation. A systematic understanding of plant systems and their mechanistic underpinnings is needed to improve crops, and this relies on our ability as a field to produce high quality proteomics resources and careful genetic evaluation using technologies like CRISPR/Cas9-mediated gene editing. As a temperate grass model, *B. distachyon* provides a framework for future studies on upstream environment-originating signaling and downstream stomatal effectors, and can contribute to improvements in agriculturally significant temperate grass crops like wheat and barley.

## Materials and methods

### Plant material and growth conditions

The Brachypodium (*B. distachyon*) line Bd21-3 was used as the wild-type background in all experiments (Vogel and Hill, 2008). Plants were generally grown as specified in Raissig *et al*. (2016) and Raissig *et al*. (2017). Briefly, seeds were sterilized for 15 min in a solution of 20% bleach and 0.1% Tween (v/v), and stratified on ½-strength Murashige and Skoog (MS) agar plates (Cassion Labs, 1% agar, pH 5.7) for at least 2 days at 4°C in dark before being transferred to 22°C chamber with a 16-h light/8-h dark cycle (110 μmol m^−2^ s^−1^). Plants on soil were grown in 4 × 4-in. pots in a greenhouse with a 20-h light/4-h dark cycle (250–300 μmol m^−2^ s^−1^; day temperature = 28°C, night temperature = 18°C), with the exception of lines *sid/bdmute-1; BdMUTE_pro_:YFP-BdFAMA* and *wild type; BdMUTE_pro_:YFP-BdFAMA*, which were grown on soil in a Percival growth room, model AR-1015L3, at 26°C illuminated with fluorescent lights (Philips F32T8/TL741 series 700) with a 16-h light/8-h dark cycle (250 μmol m^−2^ s^−1^). The *bdmute* mutant used in this study corresponds to the *sid/bdmute-1* allele from Raissig *et al*. (2017). Columbia (Col-0) was used as the wild-type background for all Arabidopsis (*A. thaliana*) experiments, except for *atmute*/+, which is the segregating G → A mutant allele from an EMS mutagenized population used in Pillitteri *et al*. (2007). *Atfama*/+ is the segregating T-DNA insertion line Salk_100073. Arabidopsis seeds were surface sterilized and grown on ½-MS plates, as described above. Arabidopsis plants grown on soil were grown in 22°C chambers as above, then transferred after flowering to light racks, all with 16-h light/8-h dark cycle (110 μmol m^−2^ s^−1^) using Philips F32T8/TL741 series 700 bulbs.

### Generation of DNA constructs

The following constructs for use in Brachypodium were generated in this study: *BdFAMA_pro_:YFP-BdFAMA*, *bdfama Cas9-g4, Ubi_pro_:YFP-BdFAMA, Ubi_pro_:BdFAMA, BdFAMA_pro_:3xYFP, BdMUTE_pro_:YFP-BdFAMA*. To create *BdFAMA_pro_:YFP-BdFAMA*, a ∼4.7-kb sequence upstream of the *BdFAMA* gene (Bradi2g22810; primers primMXA7 and primMXA8) was cloned into pIPKb001t (Raissig *et al*., 2016). Separately, the *BdFAMA* genomic sequence (primers primMXA5 and primMXA6) was cloned into pENTR (pENTR/D-TOPO Cloning Kit, Invitrogen, K240020) with a poly-alanine linker (annealed primers Ala_linker-F and Ala_linker-R) and an AscI-flanked *Citrine* YFP inserted 3′ of the gene by AscI digest. Site-directed mutagenesis with primers primMXA20 and primMXA21 was used to eliminate a NotI recognition site in the second exon that interfered with the cloning of BdFAMA. Finally, the entry clone was recombined into the pIPKb001t vector. *BdFAMA_pro_:3xYFPnls* was created as above, except with the *BdFAMA* genomic sequence substituted with 3xYFPnls pENTR. *Ubi_pro_:YFP-BdFAMA* and *Ubi_pro_:BdFAMA* were made using the monocot overexpression gateway vector pIPK002 (Himmelbach *et al*., 2007) as a backbone. *BdMUTE_pro_:YFP-BdFAMA* was created by recombining pENTR_YFP-BdFAMA with the destination vector pIPKb001_BdMUTEpro used in Raissig *et al*. (2017). We created the CRISPR constructs using the vectors pH-Ubi-cas9-7 and pOs-sgRNA (vectors and protocol described in Miao *et al*., 2013). The online server tool CRISPR-P was used to identify candidate spacer sequences (Lei *et al*., 2014). Spacers were generated by annealing oligo duplexes primMXA3 and primMXA4 for *BdFAMA* CRISPR_sgRNA_4, which, along with pH-Ubi-cas9-7, were transformed into Bd21-3 or *bdmute* to generate *bdfama-*Δ*2* and *bdmute*; *bdfama,* respectively. Primers primMXA 22-FWD and primMXA 24-REV were used to genotype the area flanking the predicted cut site.

The following constructs were generated for use in Arabidopsis lines: *AtFAMA_pro_:YFP-BdFAMA, AtFAMA_pro_:AtFAMA-YFP, AtMUTE_pro_:YFP-BdFAMA, AtMUTE_pro_:NLS-mTurq*, and *AtFAMA_pro_:NLS-mTurq*. Constructs for Arabidopsis transformation were created using Gateway LR recombination (Gateway LR Clonase II Enzyme mix, Invitrogen, 11791020). R4pGWB501 (Nakagawa *et al*., 2008), pDONR_L1_AtMUTEpro_R4 (Raissig *et al*., 2017), or pJET_L4_AtFAMApro_R1 (∼2.4 kb upstream of *AtFAMA* start codon (Wengier *et al*., 2018), and pENTR_YFP-BdFAMA were recombined to form constructs *AtMUTE_pro_:YFP-BdFAMA* and *AtFAMA_pro_:YFP-BdFAMA*, respectively. *AtFAMA_pro_:AtFAMA-YFP* was created by recombining R4pGWB640 (Nakamura *et al*., 2010), pJET_L4_AtFAMApro_R1, and pENTR_L1_gAtFAMA_L2 (cloned from gDNA with primers gFAMA-fw and gFAMA-rev and recombined with pENTR/D-TOPO). *AtMUTE_pro_:AtFAMA-Ven* was acquired from Smit *et al*. (2022). The GreenGate cloning system (Lampropoulos *et al*., 2013) was used to make Arabidopsis transcriptional markers *AtMUTE_pro_:NLS-mTurq* and *AtFAMA_pro_:NLS-mTurq* with sulfadiazine (sulf) resistance in the *pGreen0229* backbone (Hellens *et al*., 2000). All primers are listed in Supplemental Table 1.

### Generation of transgenic plant lines

Transgenic lines were created from *Brachypodium calli* derived from Bd21-3 and *bdmute* parental plants, transformed with *Agrobacterium tumefaciens* strain AGL1, selected for hygromycin resistance, and regenerated according to standard Brachypodium tissue culture protocols (http://jgi.doe.gov/our-science/scienceprograms/plant-genomics/brachypodium/). Plants regenerated from calli are referred to as T0 plants, and their immediate progeny as T1. Leaves from T0 regenerants bearing a fluorescent reporter were stained with propidium iodide (PI; 1:100 dilution of 1 mg ml^−1^ stock; Molecular Probes, Invitrogen Detection Technologies) and visually inspected using confocal microscopy for expression of the reporter as confirmation of successful transformation. The floral dip protocol with *Agrobacterium* strain GV3101 was used to transform Arabidopsis plants of Col-0, *atfama*/+, or *atmute*/+ backgrounds (Clough and Bent, 1998). *Atmute; AtMUTE_pro_:AtFAMA-Ven* was made by crossing *AtMUTE_pro_:AtFAMA-Ven* (line from Margot Smit, personal communication) into the *atmute*/+ mutant background using standard procedures (Weigel and Glazebrook, 2006). All lines are listed in Supplemental Table 2.

### Microscopy and phenotypic analysis

All Brachypodium and Arabidopsis confocal imaging was done using a Leica SP5 or Stellaris confocal microscope as described in Raissig *et al*. (2016). Briefly, the youngest emerging leaves were carefully excised with forceps from the surrounding sheath and stained in PI (1:100 dilution of 1 mg ml^−1^ stock; Molecular Probes, Invitrogen Detection Technologies) to visualize cell walls and were mounted in water to image the abaxial leaf surface. For imaging done for quantification of the nuclear signal intensity of *BdMUTE_pro_:YFP-BdFAMA*, we used a Leica SP5 equipped with an argon laser at 20% power with collection bandwidths 519–557 and 571–638 nm for PI and YFP detection, respectively; the intensity was 8% power and 200% gain for PI and 10% power and 200% gain for YFP; the same confocal laser power and settings were used across all samples. In our Brachypodium lines, reporter expression levels and cell size change depending on the stomatal developmental stage, but within early developmental stages (after asymmetric division and before SC recruitment), cell shape is relatively consistent; thus, we standardized the quantification by only measuring the fluorescent intensity in the nuclei of cells whose length and width fell between 4 and 6 μm. FIJI was then used to SUM-project still images and measure the integrated density (IntDent measurement in FIJI) of YFP nuclear signal intensity in relative fluorescence units (Schindelin *et al*., 2012). For the Stellaris confocal imaging, a white light laser was used with the following settings: YFP collection at 523–558 nm, power 5%, gain 200%; mTurq collection at 473–499 nm, power 10%, gain 200%; and PI/mcherry collection at 597–651 nm, power 2%, gain 50%. To observe the fluorescent signal in Arabidopsis myrosin cells, young true leaves were collected and stained in PI (as above) for 15 min, and firmly pressed when mounted with a glass coverslip, abaxial side up, in water.

For all differential interference contrast (DIC) imaging, either a Leica DM6 B microscope or a Leica DMi8 inverted microscope was used. To visualize tissue for DIC imaging and remove chlorophyll, Brachypodium leaves were collected and placed in a 7:1 ethanol:acetic acid solution overnight at minimum, rinsed with water, and mounted in Hoyer's medium (7.5 g gum arabic, 5 ml glycerine, 100 g chloral hydrate, 30 ml H_2_O; Vermon and Meinke, 1994). The same was done with Arabidopsis tissue, except the tissue was left in the solution for at least 2 h before rinsing and mounting to clear. Quantification of cell numbers from cleared tissue was done with images captured on the inverted Leica DMi8 microscope using a 0.29 mm^2^ field of view; in general, 5 fields of view per leaf and 6 leaves total from 6 different individuals (1 leaf per individual) were used; see figure legends for specific numbers. We sampled the fields of view from the mid region of the leaf, as stomatal spacing can be highly variable along the base and tip of the leaf. To visualize the leaf morphology of regenerants in BdFAMA overexpression lines, regenerants still attached to calli were visually inspected using a dissecting microscope (VWR Vistavision, item #26412).

### Coomassie Brilliant Blue staining of myrosin cells

To visualize mature myrosin cells in Arabidopsis, true leaves of 10- to 15-day-old plants were stained as described in Ueda *et al.* (2006): leaves were boiled for 3 min in Coomassie Brilliant Blue (CBB) solution (45% methanol, 10% acetic acid, and 0.25% CBB R250), and left to de-stain for 2–4 h in Hoyer's medium. After de-staining, tissue was mounted on glass slides with 60% glycerol and the abaxial surface was examined with DIC microscopy for myrosin cells.

### DNA extraction and reverse transcription quantitative PCR (RT-qPCR)

When DNA was extracted for genotyping using the cetyl trimethylammonium bromide DNA extraction protocol (Allen *et al*. 2006), tissue was harvested and flash-frozen with glass beads in liquid nitrogen, and ground using a Spex Certiprep Geno Grinder 2000. Alternatively, DNA was extracted using the Phire™ Plant Direct PCR Master Mix Kit (ThermoScientific) following the manufacturer's instructions, using ∼2–5 mm diameter tissue sample obtained from a young leaf suspended in 50 μl of the provided dilution buffer and either ground with the tip of a pipette or boiled for 10 min. Sanger sequencing was used following PCR for genotyping; genotyping primers can be found in Supplemental Table 1. We extracted RNA for use in RT-qPCR from wild-type, *bdmute*, and *bdfama* leaves from the division zone (3 individuals were included per replicate, with 3 replicates, except for *bdfama* which had 2 individuals per replicate; the first leaf was removed and ∼4 mm of the base of emerging leaves, starting from the shoot apical meristem, were sampled) using the RNeasy Mini kit (Qiagen) following the manufacturer's instructions, including optional DNase treatment. To produce single-stranded cDNA, we used the iScript™ cDNA Synthesis Kit (Bio-Rad) and quantified the cDNA using a Nanodrop (ThermoScientific, NanoDrop 1^c^). For RT-qPCR, we used the SsoAdvanced™ Universal SYBR^®^ Green Supermix **(**Bio-Rad) and a CFX96™ Real Time System (Bio-Rad) running a standard program (initially 95°C for 30 s, then 40 cycles of 95°C for 10 s and 60°C for 30 s, followed by melting curve analysis). Expression values were normalized to control gene *BdUBC18* and are relative to the expression of wild-type plants using the 2^−ΔΔ*CT*^ method. All RT-qPCR primers were designed using QuantPrime (www.quantprime.de). Primer sequences can be found in Supplemental Table 1.

### Protein alignments

Protein alignment of BdFAMA (Bradi2g22810), BdMUTE (Bradi1g18400), AtFAMA (AT3G24140), and AtMUTE (AT3G06120) was done using the Clustal 2.1 multiple sequencing alignment (MSA) tool (https://www.ebi.ac.uk/Tools/msa/). Brachypodium sequences were obtained from the *B. distachyon v3.1* in Phytozome 10. Protein domains were identified using the National Center for Biotechnology Information conserved domain search tool (https://www.ncbi.nlm.nih.gov/Structure/cdd/wrpsb.cgi).

### Yeast two-hybrid (Y2H) assays

To obtain cDNA suitable for creating Y2H-compatible Brachypodium genes, Bd21-3 seedlings were grown on ½-MS plates and the bottom 5 mm (developmental zone) of young true leaves were harvested, flash-frozen, and ground for RNA extraction using the RNeasy Plant MiniKit (Quiagen), followed by reverse transcription with SuperScript IV reverse transcriptase (Thermo Fisher) according to the manufacturer's instructions. Genes with stop codons were amplified using the primers in Supplemental Table 1 and cloned into pENTR/D-TOPO, followed by LR recombination with Gateway-compatible pGAD-T7 and pGBK-T7 (Clontech). For Y2H assays, bait and prey plasmids were transformed into the yeast strain AH109, followed by the selection of transformants and testing of pairwise interactions by growth complementation assays on nutrient selection media as described in the Matchmaker™ GAL4 Two-Hybrid System 3 manual (Clontech; Takara Bio USA, Inc, 2009). Three replicates per pairwise interaction were tested, and representative images were used for the figures. To compensate for the auto-activation of some constructs, plates containing 10–30 mM 3-amino-1,2,4-triazole (3-AT) were included in the interaction assays.

### Co-IP–LC-MS/MS

#### Plant lines

Wild-type Brachypodium Bd21-3 and the following published reporter lines in the wild-type background were used for co-IPs: *BdSPCH2_pro_:BdSPCH2-CitYFP* (Raissig *et al*., 2016), *BdMUTE_pro_:CitYFP-BdMUTE* (construct and transformation strategy described in (Raissig *et al*., 2017)), *BdSCRM2_pro_:CitYFP-BdSCRM2* (Raissig *et al*., 2016), *ZmUBI1_pro_:YFP-BdICE1* (Raissig *et al*., 2016), *BdMUTE_pro_:3xYFPnls* (Raissig *et al*., 2017). The creation of *BdFAMA_pro_:YFP-BdFAMA* is described above.

#### Growth and harvesting conditions

For each sample, about 100 seeds were surface sterilized with 1% (v/v) bleach, 0.1% (v/v) Tween-20 for 10 min, washed thoroughly with water, stratified on ½-MS plates (Caisson labs, 1% (w/v) Agar, pH 5.7) for 4–6 days at 4°C and transferred to a growth chamber set to long-day conditions (16 h/8 h light/dark, 22°C). Starting from the second leaf until the fifth or sixth main shoot leaf, all emerging leaves were gently pulled off the plant and the bottom 4 mm containing the developmental zone (as determined by microscopy) were harvested, flash-frozen in liquid nitrogen, and stored at −80°C until further use. Plants were returned to the chamber to grow additional leaves after each round of harvesting. In total, about 150–220 mg of plant materials from 600 to 700 leaves were obtained per sample.

#### Affinity purification

For each line, three biological replicates were used. Frozen plant material was ground to a fine powder in a ball mill, weighed, and resuspended in 5 µl mg^−1^ extraction buffer (25 mM Tris pH 7.5, 10 mM MgCl_2_, 0.5 mM EGTA, 75 mM NaCl, 1% (v/v) Triton X-100, 1 mM NaF, 0.5 mM Na_3_VO_4_, 15 mM β-glycerophosphate, 1 mM DTT, 1 mM PMSF, 1× complete proteasome inhibitor) by rotating at 4°C for 15 min. Cells were ruptured by 5 × 15 s sonication in an ice bath in a Bioruptor UCD-200 (Diagenode) at medium intensity with 2 min breaks on ice. To further break cell walls and digest DNA, 2 µl Lysonase (Millipore) was added, and samples were incubated at 4°C for 30 min with endo-over-end rotation. The extracts were cleared by 10 min centrifugation at 11,750*×g* and the supernatant was incubated on a rotor wheel at 4°C for 3 h with 25 µl of GFP-Trap magnetic agarose bead slurry (Chromotek), and pre-washed with extraction buffer. The beads were washed tree times with 500 µl wash buffer (extraction buffer with 100 mM NaCl and without proteasome inhibitor), resuspended in 50 µl 2× Laemmli buffer, boiled for 5 min at 95°C, and frozen at −80°C until further processing. Successful affinity purification was confirmed by immunoblotting using rat monoclonal anti-GFP antibody (3H9, Chromotek, diluted 1:2,000 in 5% [w/v] skim milk powder dissolved in tris buffer saline with 0.1% [v/v] Tween-20 [TBS-T]) and AffiniPure Donkey Anti-Rat IgG-HRP (712-035-153, Jackson Immuno Research Laboratories, diluted 1:10,000 in the same solution).

#### Sample prep and mass spectrometry

For MS analysis, purified proteins were briefly separated by SDS-PAGE on Mini PROTEAN TGX gels (Bio-Rad), stained with the Novex colloidal blue staining kit (Invitrogen), and excised and further processed by in-gel Tryptic digestion. Peptides were desalted using C18 ZipTips (Millipore) and resuspended in 0.1% formic acid. LC-MS/MS was performed on a Q-Exactive HF hybrid quadrupole-Orbitrap mass spectrometer (Thermo Fisher) with an Easy LC 1200 UPLC liquid chromatography system (Thermo Fisher) and analytical column ES803 (Thermo Fisher). Peptides were eluted with the following conditions at a flow rate of 300 nl min^−1^: a 100-min gradient from 5% to 28% solvent B (80% [v/v] acetonitrile, 0.1% [v/v] formic acid), followed by a 20-min gradient from 28% to 44% solvent B and a short wash with 90% solvent B. Precursor scan was from mass-to-charge ratio (*m/z*) 375 to 1,600 and the top 20 most intense multiply charged precursors were selected for fragmentation with higher-energy collision dissociation (HCD) with normalized collision energy (NCE) 27.

#### Data analysis

Protein identification and label-free quantification (LFQ) were done in MaxQuant (version 1.6.2.6; Tyanova *et al*., 2016a) using the default settings with the following modifications: LFQ minimum ratio count was set to 1, “Fast LFQ” and “Match between runs” were enabled. Peptides were searched against the *B. distachyon* Bd21-3 protein database (v1.1) obtained from Phytozome containing a total of 47,917 entries (https://phytozome-next.jgi.doe.gov/info/BdistachyonBd21_3_v1_1) plus a list of likely contaminants containing Trypsin, human Keratin, and YFP and against the contaminants list of MaxQuant. Search results were then analyzed in Perseus (version 1.6.2.3; Tyanova *et al*., 2016b). LFQ intensities were imported, and proteins marked as “only identified by site”, “reverse”, “potential contaminant” and, such, that were not identified in at least two replicates of one sample group were removed. Data were log2 transformed and missing values were imputed from a normal distribution (width = 0.3, down shift = 1.8, total matrix mode). Significantly enriched proteins were identified by unpaired two-tailed Student's *t*-tests (modified permutation-based FDR with 250 randomizations for multiple sample correction; S0 = 0.5) comparing the bHLH reporter lines with either of the two control lines. Only proteins that were identified in at least two replicates of the bHLH reporter line were used for the test. False discovery rates (FDRs) were chosen to get a minimum number of false negatives: 0.015 for BdSPCH2 versus Bd21-3, 0.025 for BdMUTE/BdICE1/YFP versus Bd21-3, 0.05 for BdFAMA/BdSCRM2 versus Bd21-3, 0.075 for BdSPCH2/BdMUTE/BdFAMA/BdSCRM2 versus YFP, and 0.04 for BdICE1 versus YFP and 0.035 for Bd21-3 versus YFP. To be considered bHLH interaction candidates, proteins had to meet the following criteria: (1) they had to be identified by at least one MS/MS spectrum in the respective sample group, (2) they had to be significantly enriched compared with at least one of the controls, and (3) the fold change in the bHLH samples had to be at least 1.5 times higher than that of the other control or 2 times if the other control was also significantly enriched. Data were also searched with Protein Prospector using the same databases and search parameters described in (Shrestha *et al*., 2022). Search results were compared using a protein and peptide FDR cutoff of 5% and 1% with “Keep Replicates” enabled. Peptide counts of candidates are largely in agreement with MaxQuant results.

OrthoFinder (Emms and Kelly, 2019) and the Brachypodium gene IDs (circa 2019) were used to identify putative orthologs of candidate proteins in other plant species. Gene ontology (GO) terms (biological processes) for rice and Arabidopsis genes were obtained from UniProt. Additional GO analysis of the Brachypodium candidates and their best rice and Arabidopsis putative orthologs was done with AgriGO v2.0 (Tian *et al*., 2017). Likely protein function was deduced from GO term analysis and functional annotation on TAIR (Arabidopsis.org). Hierarchical clustering and principal component analysis (PCA) were done in Perseus. PCA, scatter plots, and Venn diagrams were plotted in RStudio (RStudio Team, 2022). The MS data (raw files and search results) have been deposited to the ProteomeXchange Consortium via the PRIDE [1] partner repository (http://proteomecentral.proteomexchange.org) with the data set identifier PXD035582.

### Statistical analysis and plotting

Statistical analysis was performed in R. For phenotypic quantifications, two sample tests (i.e. wild-type line versus mutant line, or reporter line A fluorescence versus reporter line B fluorescence) were performed using the Wilcoxon rank sum test followed by Dunn's multiple comparisons test to correct for multiple sampling error. To analyze RT-qPCR data, Welch's *t*-test was applied to compare expression in the mutant line relative to the wild-type. Statistical data are provided in Supplemental Data Set 2.

### Accession numbers

Sequence data for genes discussed in this article can be found at PHYTOZOME v.12 for *A. thaliana* (TAIR10), *B. distachyon* (v.3.1 JGI), *O. sativa* (rice; v.7 JGI), and from PLAZA 3.0 Monocots for *Z. mays* under the accession numbers Bradi2g22810 (*BdFAMA*), Os05g50900 (*OsFAMA*), AT3G24140 (*AtFAMA*), Zm08g11540 (*ZmFAMA,* putative), AT3G06120 (*AtMUTE*), Bradi1g18400 (*BdMUTE*), Os05g51820 (*OsMUTE*), Zm08g12070 (*ZmMUTE*), AT5G53210 (*AtSPCH*), Bradi1g38650 (*BdSPCH1*), Bradi3g09670 (*BdSPCH2*), AT1G12860 (*AtSCRM2*), AT3G26744 (*AtICE1*), Bradi4g17460 (*BdICE1*), and Bradi2g59497 (*BdSCRM2*).

## Supplemental data

The following materials are available in the online version of this article.

**Supplemental Figure 1.** Characterization of BdFAMA mutations and mutant phenotypes.

**Supplemental Figure 2.** Additional phenotypes of bdmute; BdFAMApro:YFP-BdFAMA and bdmute; BdMUTEpro:YFP-BdFAMA.

**Supplemental Figure 3.** Dose-dependent phenotypes of bdmute; BdMUTEpro:YFP-BdFAMA and WT; BdMUTEpro:YFP-BdFAMA.

**Supplemental Figure 4.** Expression patterns of Brachypodium stomatal bHLH transgenes used in co-IP experiments.

**Supplemental Figure 5.** Bait enrichment in the co-IP.

**Supplemental Figure 6.** Summary of proteins enriched in each stomatal bHLH co-IP experiment.

**Supplemental Figure 7.** Summary of unique and shared putative interaction partners from co-IP assays of stomatal bHLHs.

**Supplemental Figure 8.** Y2H confirms bHLH interactions identified by co-IP assays.

**Supplemental Figure 9.** Protein sequence alignments of MUTE and FAMA in Brachypodium and Arabidopsis.

**Supplemental Figure 10.** Additional phenotypes revealed in rescue experiments with Arabidopsis and Brachypodium stomatal bHLHs.

**Supplemental Table 1.** Primer sequences used in this study.

**Supplemental Table 2.** Summary of lines used in this study.

**Supplemental Data Set 1**. Candidate interactors of Brachypodium stomatal bHLHs from co-IPs (.xls).

**Supplemental Data Set 2**. Statistical information.

## Supplementary Material

koac341_Supplementary_Data
