# Supplementary material for: Expanded roles and divergent regulation of FAMA in Brachypodium and Arabidopsis stomatal development
Source: Plant Cell. 2022 Nov 28;35(2):756–75. doi: 10.1093/plcell/koac341 (PMC9940870; doi:10.1093/plcell/koac341)
Supplement: koac341_Supplementary_Data [file koac341_supplementary_data.zip › tpc.22.00575_Supplemental.pdf]

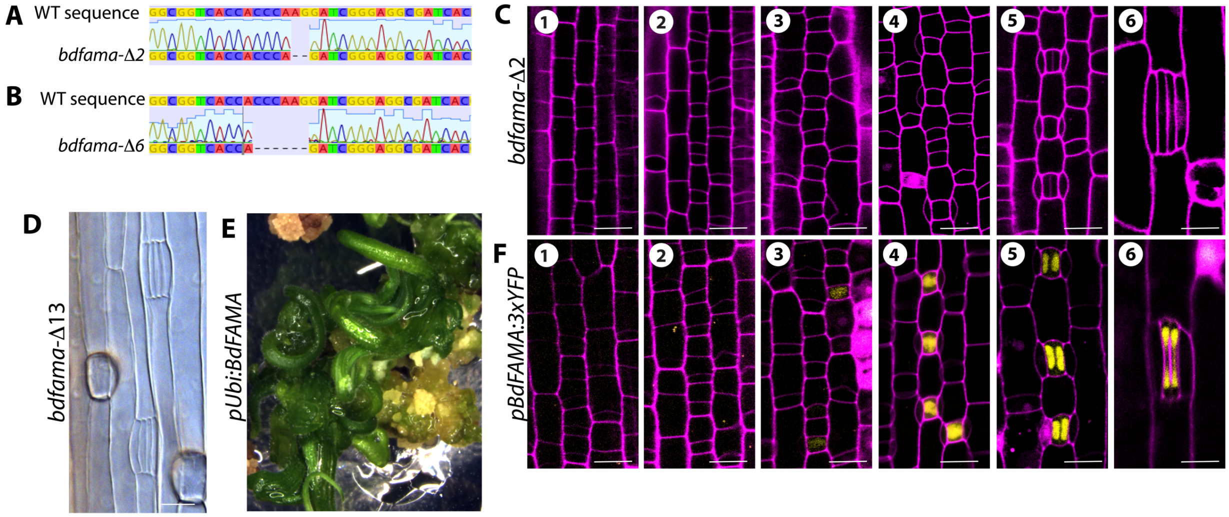

Figure S1. Characterization of *BdFAMA* mutations and mutant phenotypes. (A-B) Chromatograms of *bdfama-Δ2* (null mutant) and *bdfama-Δ6* (in-frame deletion with wild-type phenotype). **(C)** Confocal images of developmental stages of *bdfama-Δ2* during stomatal file establishment (stage 1), asymmetric division (stage 2), subsidiary mother cell (SMC) establishment (stage 3), subsidiary cell (SC) recruitment (stage 4), guard cell (GC) division (stage 5), and mature stomatal complexes (stage 6). **(D)** Differential interference contrast (DIC) images of cleared tissue from homozygous mutant T0 regenerant with a 13-bp deletion, *bdfama-Δ13.* **(E)** Image of regenerants from transformation with Ubi_{pro}*:BdFAMA* showing severe morphological defects in leaf tissues (5x magnification). **(F)** Confocal images of *BdFAMA* transcriptional reporter, BdFAMA_{pro}*:3xYFP,* during development. Cell outlines (magenta) visualized by propidium iodide (PI) staining. Scale bar = 10 µm.
[Supports Figure 1]

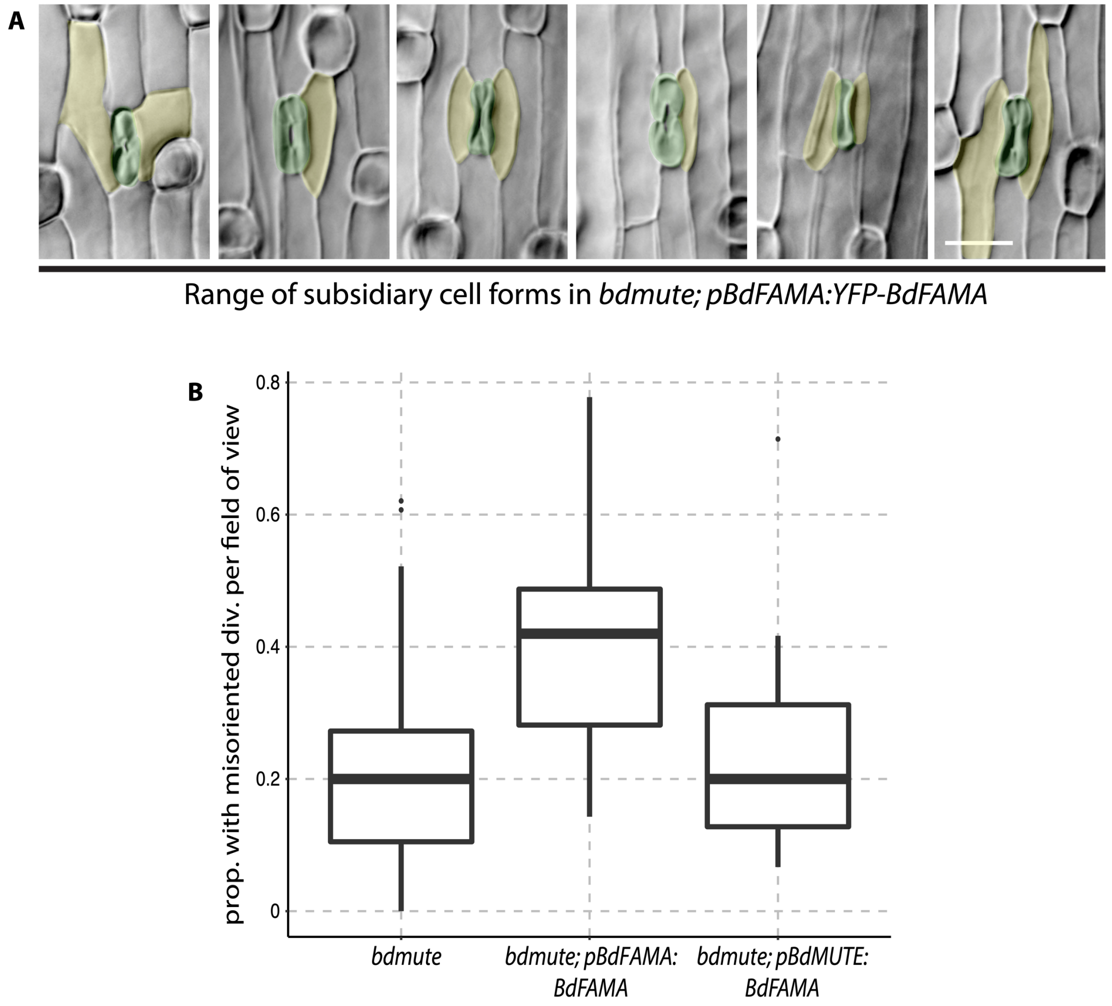

Figure S2. Additional phenotypes of *bdmute; BdFAMA$_{pro}$:YFP-BdFAMA* and *bdmute; BdMUTE$_{pro}$:YFP-BdFAMA.* (A) Representative images of the phenotypic range of subsidiary cells recruited in *bdmute; BdFAMA$_{pro}$:YFP-BdFAMA.* Guard cells and presumptive subsidiary cells are pseudo-colored in green and yellow, respectively. **(B)** Proportion of stomatal complexes with misoriented guard mother cell (GMC) divisions per 0.29 mm^2 field of view in *bdmute, bdmute; BdFAMA$_{pro}$:YFP-BdFAMA* and *bdmute; BdMUTE$_{pro}$:YFP-BdFAMA.* n=623, 2,354, and 1,349 stomata from 5 fields of view per leaf and 6 leaves, 1 leaf each from 6 different individuals for *bdmute, bdmute; BdFAMA$_{pro}$:YFP-BdFAMA* and *bdmute; BdMUTE$_{pro}$:YFP-BdFAMA,* respectively.
[Supports Figure 2]

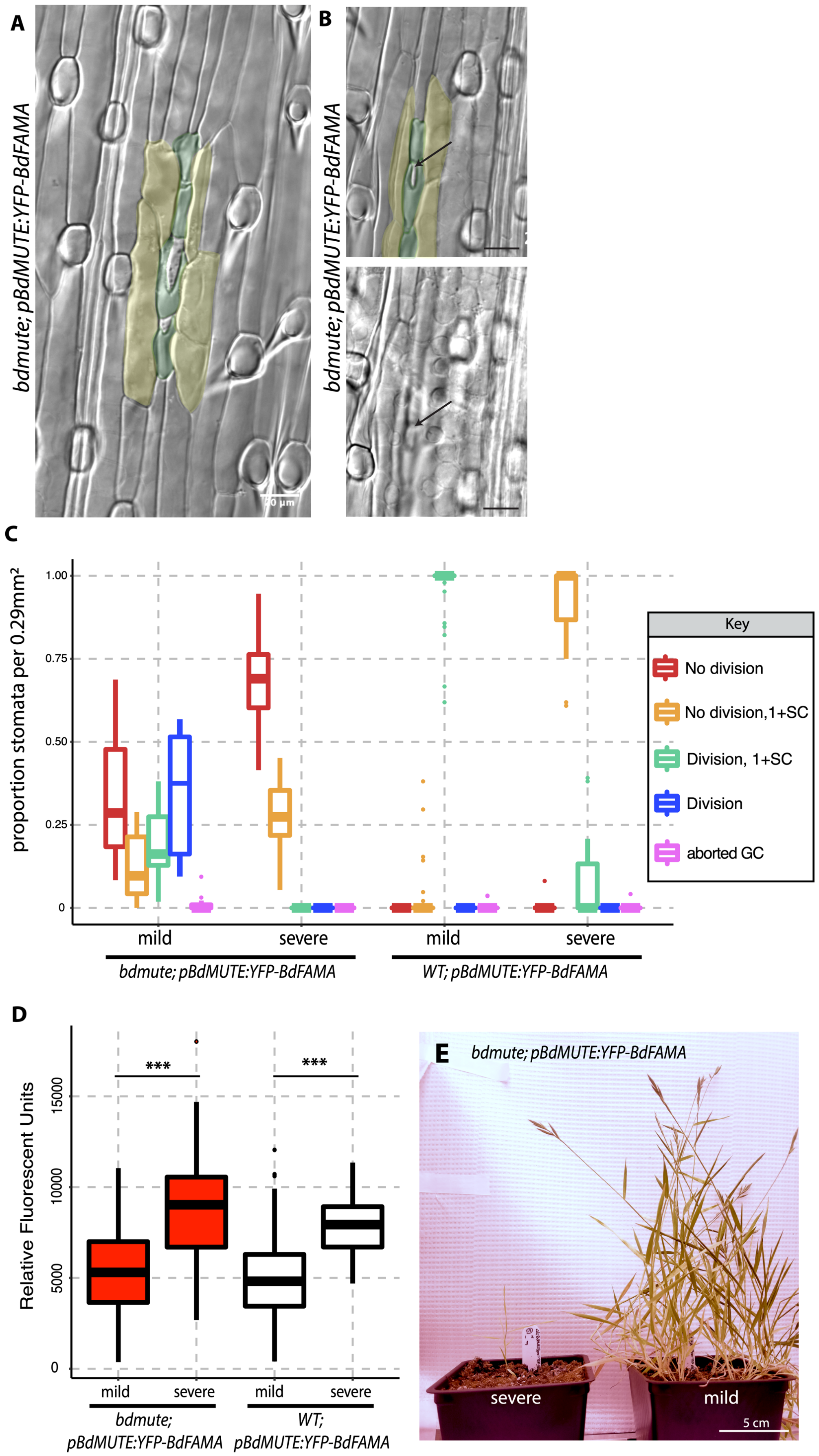

A
bdmute; pBdMUTE:YFP-BdFAMA
B
bdmute; pBdMUTE:YFP-BdFAMA
C
proportion stomata per 0.29mm²
1.00
0.75
0.50
0.25
0
Key
No division
No division,1+SC
Division, 1+SC
Division
aborted GC
mild
severe
mild
severe
bdmute; pBdMUTE:YFP-BdFAMA
WT; pBdMUTE:YFP-BdFAMA
D
Relative Fluorescent Units
15000
10000
5000
0
***
***
mild
severe
mild
severe
bdmute; pBdMUTE:YFP-BdFAMA
WT; pBdMUTE:YFP-BdFAMA
E
bdmute; pBdMUTE:YFP-BdFAMA
severe
mild
5 cm

Figure S3. Dose-dependent phenotypes of *bdmute; BdMUTE$_{pro}$:YFP-BdFAMA* and *WT; BdMUTE$_{pro}$:YFP-BdFAMA.* **(A-B)** DIC images of stomatal cell clusters in severe *bdmute; BdMUTE$_{pro}$:YFP-BdFAMA* lines that give rise to a pore. **(B)** Representative image of stomatal clusters of single guard cells that give rise to a pore with underlying mesophyll airspace. Cleared tissue from the 2nd leaf, 6–7 days post-germination in T1 *bdmute; BdMUTE$_{pro}$:YFP-BdFAMA* plants. **(C)** Quantification of stomatal phenotypes in *bdmute* and wild type (Bd21-3) lines expressing *BdMUTE$_{pro}$:YFP-BdFAMA* from cleared abaxial tissue of the 2nd leaf, 6–7 days post-germination (dpg) in T1 plants. Key (right) shows colors corresponding to phenotypic classes. For each sample, five different regions of the leaf were imaged and quantified; the same quantification of phenotypes used to generate Fig. 2C. *bdmute; BdMUTE$_{pro}$:YFP-BdFAMA,* is used here, but classified by line severity. n=1,349 stomata and 40 fields of view (8 plants) for *bdmute; BdMUTE$_{pro}$:YFP-BdFAMA* and n=1,450 stomata and 47 fields of view (10 plants) for *WT; BdMUTE$_{pro}$:YFP-BdFAMA*. **(D)** Quantification of fluorescent intensity, measured in relative fluorescence units (RFU) in severe and mild lines of *bdmute; BdMUTE$_{pro}$:YFP-BdFAMA* (red boxes) and *WT; BdMUTE$_{pro}$:YFP-BdFAMA* (white boxes) using confocal images from the same leaves used in (A). Raw microscopy images were SUM projected in FIJI and the integrated density measurement was used to approximate YFP nuclear signal. To standardize quantification, only the nuclei of cells in the developmental zone that fell between 4-6 μm in length and width were used in the analysis. n=130 cells for *bdmute; BdMUTE$_{pro}$:YFP-BdFAMA* and n=172 cells for *WT; BdMUTE$_{pro}$:YFP-BdFAMA*. ***P<0.001 (based on a Wilcoxon rank sum test). **(E)** Plant growth differences between mild and severe lines of *bdmute; BdMUTE$_{pro}$:YFP-BdFAMA.* Scale bar = 5cm.
[Supports Figure 2]

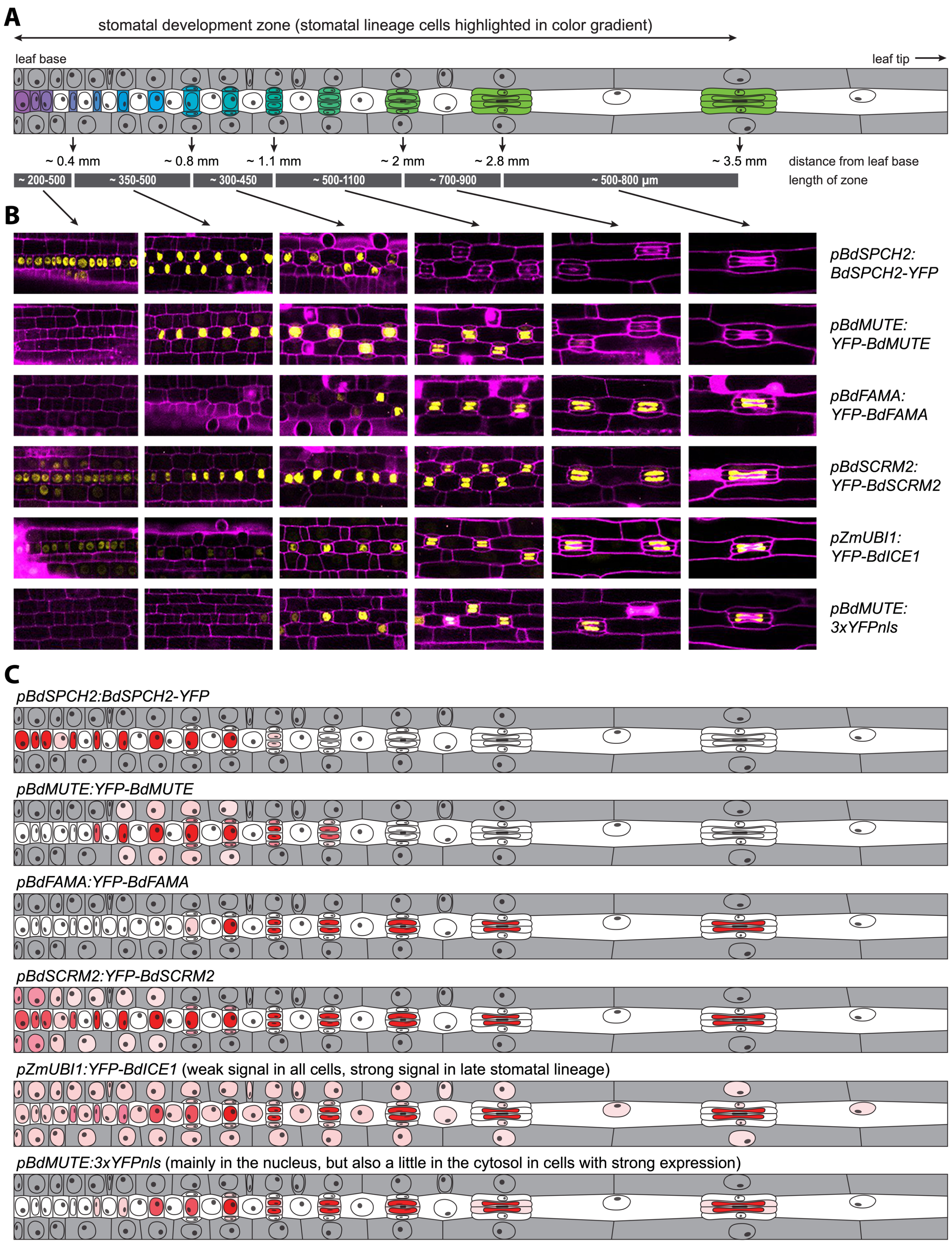
A
stomatal development zone (stomatal lineage cells highlighted in color gradient)
leaf base
leaf tip
~ 0.4 mm
~ 0.8 mm
~ 1.1 mm
~ 2 mm
~ 2.8 mm
~ 3.5 mm
distance from leaf base
~ 200-500
~ 350-500
~ 300-450
~ 500-1100
~ 700-900
~ 500-800 µm
length of zone
B
pBdSPCH2: BdSPCH2-YFP
pBdMUTE: YFP-BdMUTE
pBdFAMA: YFP-BdFAMA
pBdSCRM2: YFP-BdSCRM2
pZmUBI1: YFP-BdICE1
pBdMUTE: 3xYFPnls
C
pBdSPCH2:BdSPCH2-YFP
pBdMUTE:YFP-BdMUTE
pBdFAMA:YFP-BdFAMA
pBdSCRM2:YFP-BdSCRM2
pZmUBI1:YFP-BdICE1 (weak signal in all cells, strong signal in late stomatal lineage)
pBdMUTE:3xYFPnls (mainly in the nucleus, but also a little in the cytosol in cells with strong expression)

Figure S4. Expression patterns of Brachypodium stomatal bHLH transgenes used in co-IP experiments.

(A) Scheme showing the stages of stomatal development along the base of a young leaf. Stomatal lineage cells are highlighted with a purple-blue-green gradient. The approximate distance of each stage (first visible asymmetric cell divisions, recruitment of subsidiary cells, symmetric division of the guard mother cell (GMC), elongation of the complex, formation of the typical dumbbell shape of guard cells (GCs), complex fully elongated) from the leaf base, as well as the length of each zone as determined by confocal microscopy of different plant lines is given below. **(B)** Confocal images showing expression of indicated reporter constructs in each zone in the lines used for the experiment (yellow). Leaves were stained with propidium iodide (PI) to visualize cell outlines (magenta). **(C)** For better comparison, expression of the reporter constructs in the epidermis is also shown in the schematic diagrams with shades of red indicating the expression level within a line (red = high, pink = low). All signals were nuclear.

[Supports Figure 3]

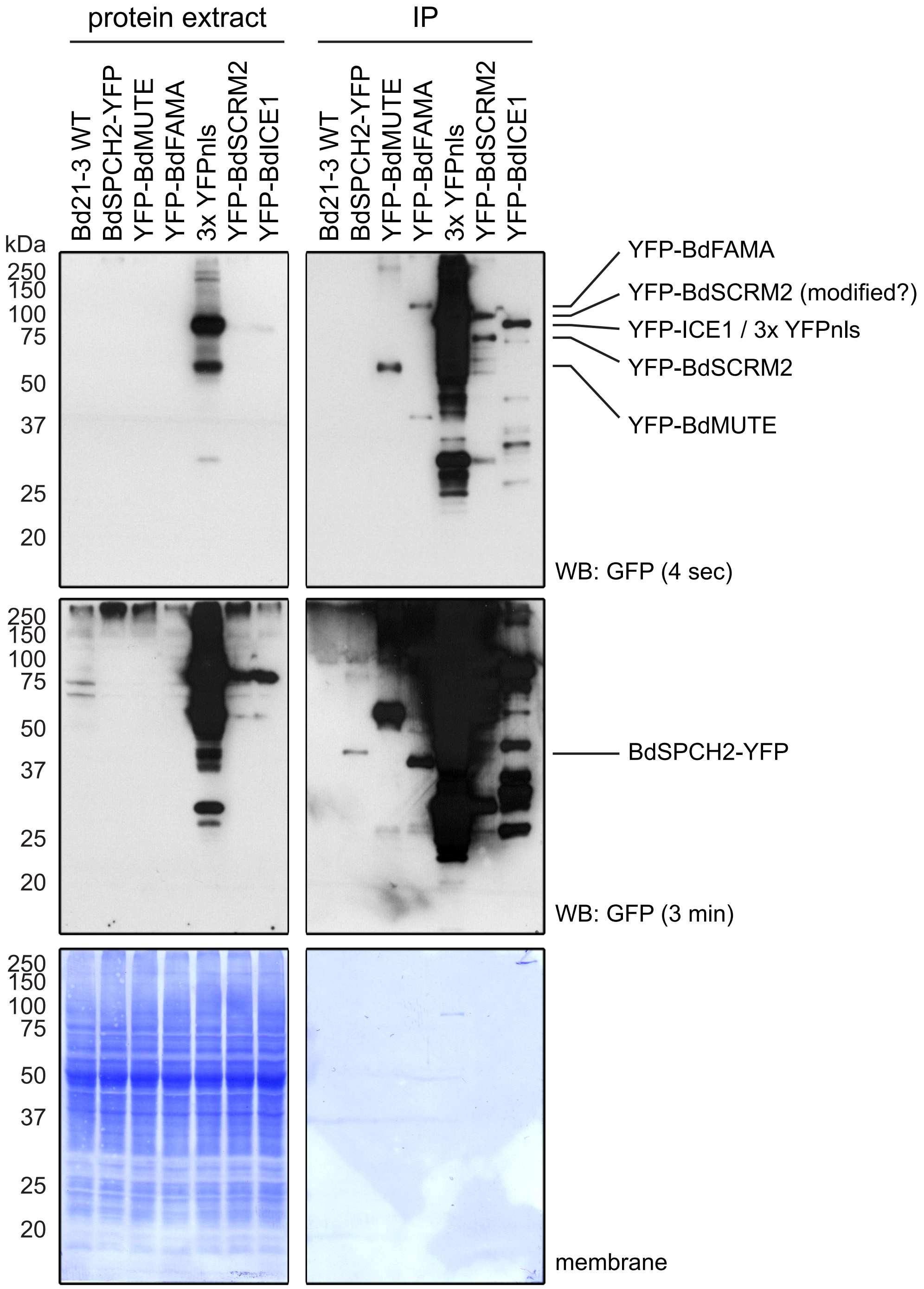

Figure S5. Bait enrichment in the co-IP. Immunoblot analysis of co-IP input and proteins eluted from the beads after IP using an anti-GFP antibody. A short (top) and long (middle) exposure, as well as the Coomassie Brilliant Blue-stained membranes (bottom) are shown. The position of the YFP fusion proteins is indicated on the right.

[Supports Figure 3]

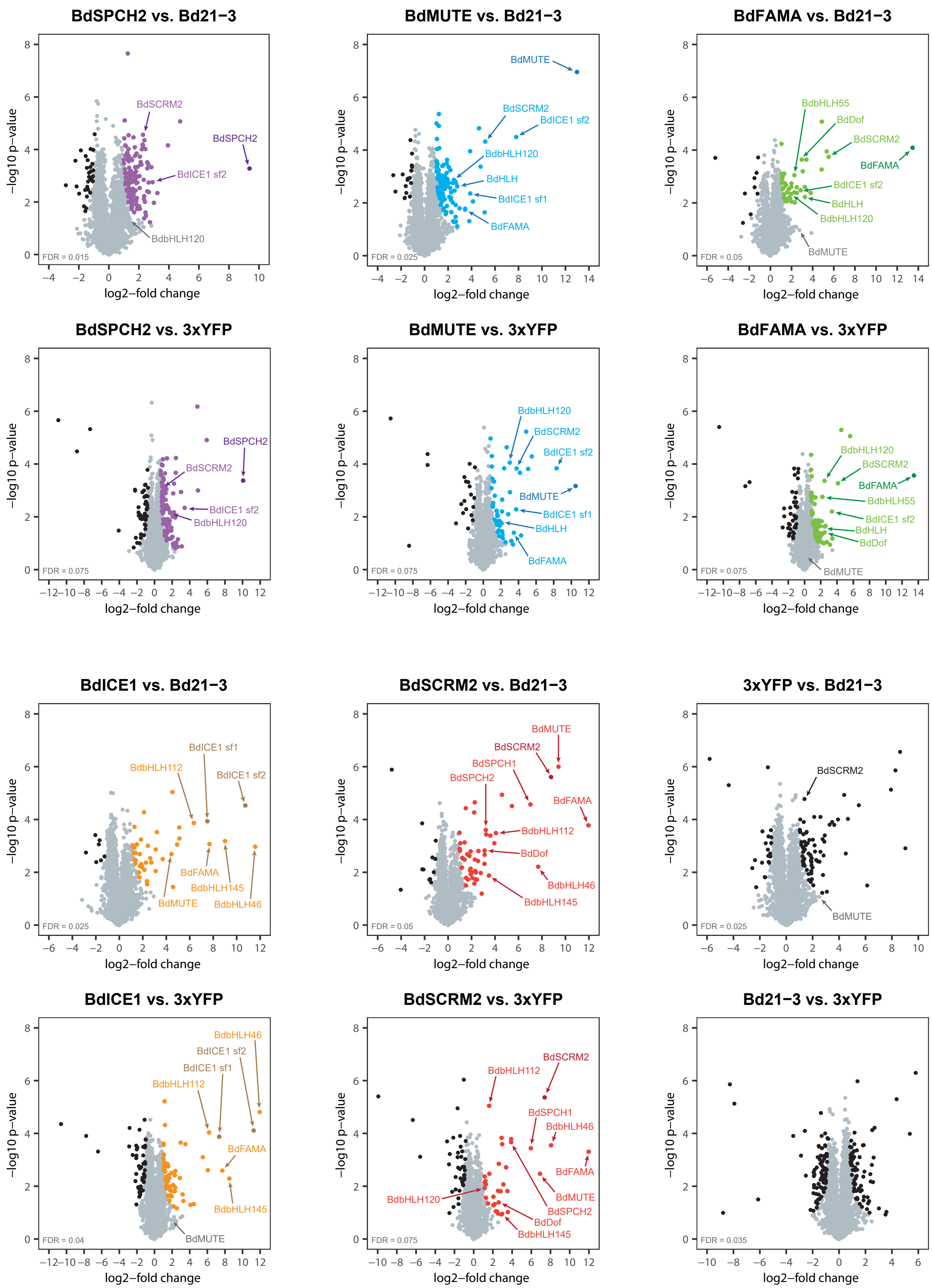
BdSPCH2 vs. Bd21−3
BdSCRM2
BdSPCH2
BdICE1 sf2
BdbHLH120
FDR = 0.015
BdMUTE vs. Bd21−3
BdMUTE
BdSCRM2
BdICE1 sf2
BdbHLH120
BdHLH
BdICE1 sf1
BdFAMA
FDR = 0.025
BdFAMA vs. Bd21−3
BdbHLH55
BdDof
BdSCRM2
BdFAMA
BdICE1 sf2
BdHLH
BdbHLH120
BdMUTE
FDR = 0.05
BdSPCH2 vs. 3xYFP
BdSPCH2
BdSCRM2
BdICE1 sf2
BdbHLH120
FDR = 0.075
BdMUTE vs. 3xYFP
BdbHLH120
BdSCRM2
BdICE1 sf2
BdMUTE
BdICE1 sf1
BdHLH
BdFAMA
FDR = 0.075
BdFAMA vs. 3xYFP
BdbHLH120
BdSCRM2
BdFAMA
BdbHLH55
BdICE1 sf2
BdHLH
BdDof
BdMUTE
FDR = 0.075
BdICE1 vs. Bd21−3
BdICE1 sf1
BdbHLH112
BdICE1 sf2
BdFAMA
BdbHLH145
BdMUTE
BdbHLH46
FDR = 0.025
BdSCRM2 vs. Bd21−3
BdMUTE
BdSCRM2
BdSPCH1
BdSPCH2
BdFAMA
BdbHLH112
BdDof
BdbHLH46
BdbHLH145
FDR = 0.05
3xYFP vs. Bd21−3
BdSCRM2
BdMUTE
FDR = 0.025
BdICE1 vs. 3xYFP
BdbHLH46
BdICE1 sf2
BdICE1 sf1
BdbHLH112
BdFAMA
BdbHLH145
BdMUTE
FDR = 0.04
BdSCRM2 vs. 3xYFP
BdSCRM2
BdbHLH112
BdSPCH1
BdbHLH46
BdFAMA
BdbHLH120
BdMUTE
BdSPCH2
BdDof
BdbHLH145
FDR = 0.075
Bd21−3 vs. 3xYFP
FDR = 0.035
−log10 p−value
log2−fold change

Figure S6. Summary of proteins enriched in each stomatal bHLH co-IP experiment. Scatter plots showing the log2-fold change and -log10 p-value from unpaired two-tailed t-tests between the bHLH reporter lines and each of the two controls with a permutation-based false-discovery rate (FDR) for multiple sample correction (S0 = 0.5). The FDR for each comparison was selected to minimize false negatives (proteins enriched in the control are highlighted in black). Proteins significantly enriched in the bHLH reporter lines are highlighted in color and the positions of candidates highlighted in this paper are indicated in the plots.

[Supports Figure 3]

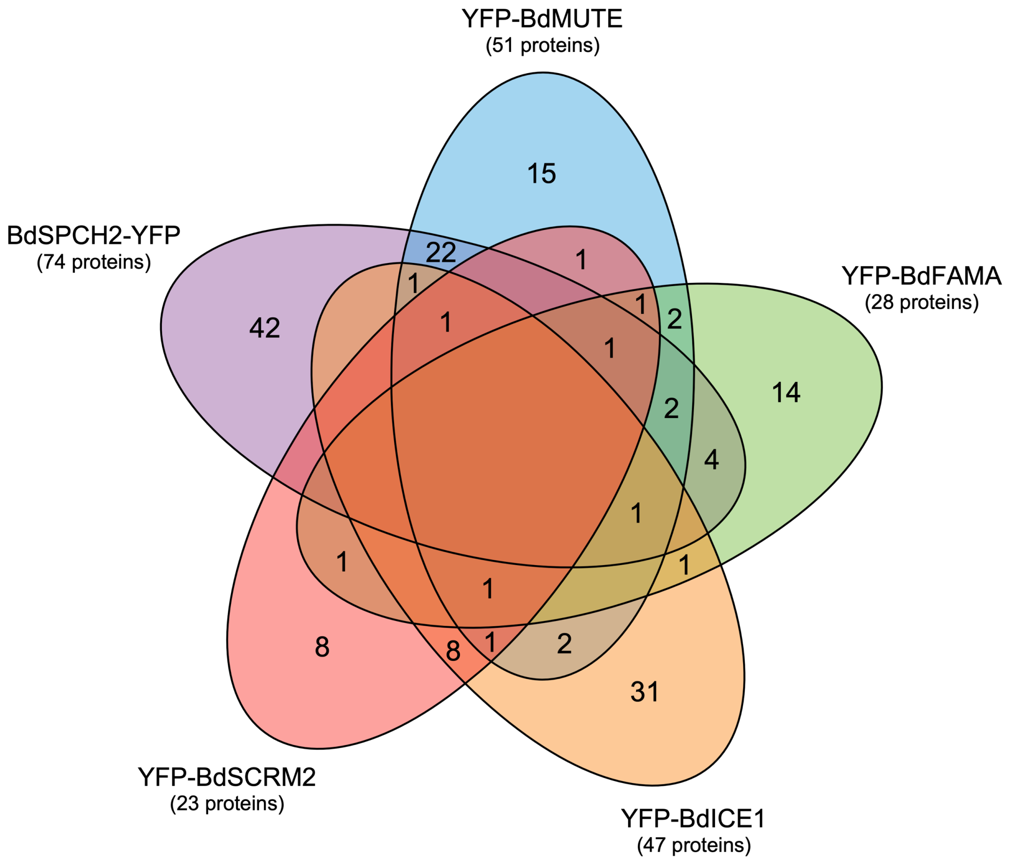

Figure S7. Summary of unique and shared putative interaction partners from co-IP assays of stomatal bHLHs. Five-way Venn diagram showing the amount of overlap of putative interaction partners of BdSPCH2 (purple), BdMUTE (blue), BdFAMA (green), BdICE1 (orange) and BdSCRM2 (red) identified by co-IP and LC–MS/MS analysis.

[Supports Figure 3]

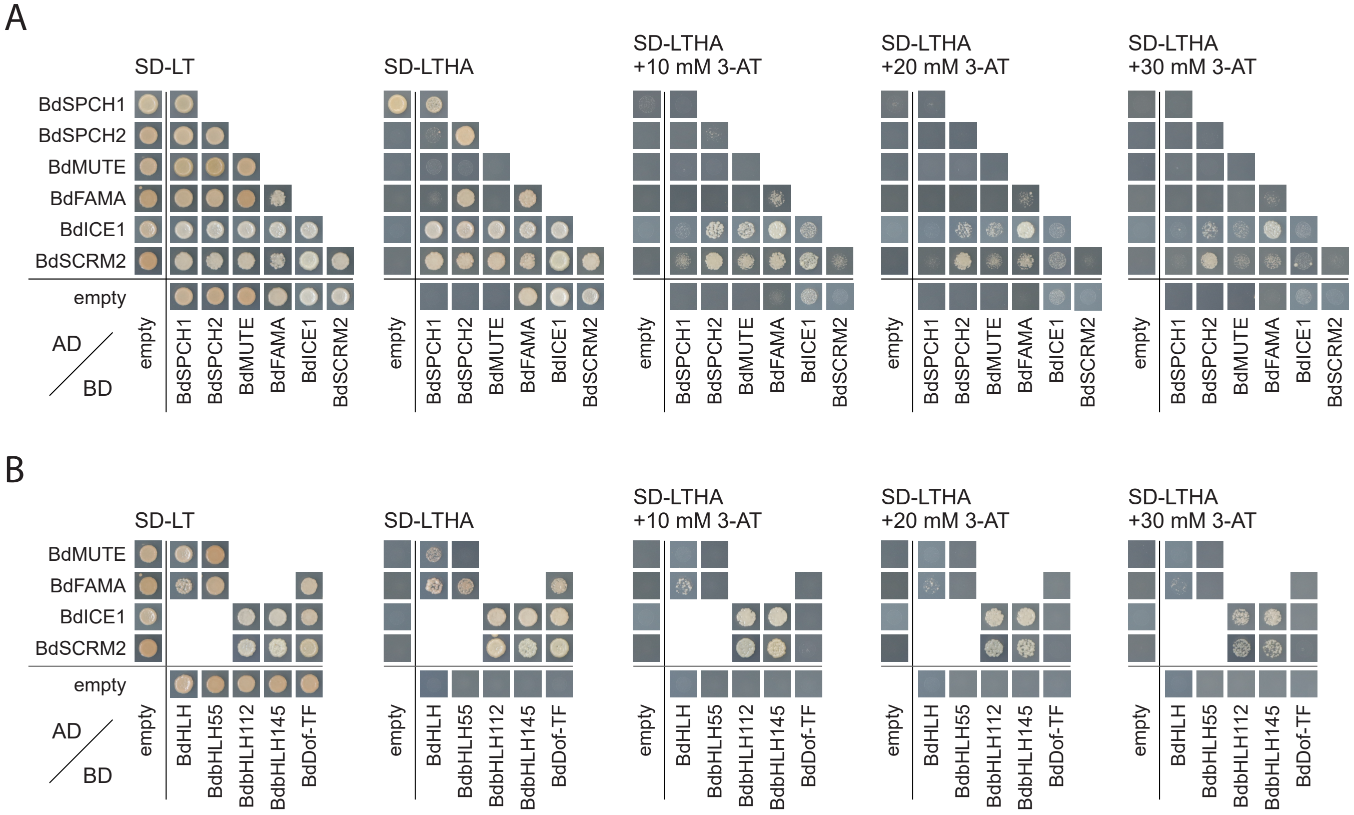

Figure S8. Y2H confirms bHLH interactions identified by Co-IP assays.
Yeast two-hybrid (Y2H) assays testing interactions between the six master regulators of stomatal development **(A)** and with selected additional transcription factors identified in the co-IP experiments. **(B)**. Yeast transfected with the indicated activation domain (AD)- and binding domain (BD)-fusion proteins was spotted on synthetic defined medium plates without leucine and tryptophane (SD-LT) or leucine, tryptophane, histidine and alanine (SD-LTHA)to test for successful transfection and protein interaction, respectively. Different concentrations of 3-AT were added to increase stringency of the selection and to overcome auto-activation of BD fusions of BdFAMA, BdICE1 and BdSCRM2. Shown is one replicate of OD = 1 suspension. Controls with the empty BD plasmid are replicated in (A) and (B).

[Supports Figure 3]

```
AT3G24140-AtFAMA      -----MDKDYSAPNFLGESSGGNDDNSSGMIDYMFNRNLQQQQKQSMPQQQQHQLSPSGF
Bradi2g22810-BdFAMA   MEKQSEQGSNNQQQQQLDSFAPLDGAAPDQDQIIGGGAGAEMVDYMLGQQTPPPPPPPHG
AT3G06120-AtMUTE      ------------------------------------------------------------
Bradi1g18400-BdMUTE   ------------------------------------------------------------


AT3G24140-AtFAMA      GATPFDKMNFSDVMQFADFGSKLALNQTRNQDDQ—G--IDPVYFLKFPVLNDKIEDHNQ
Bradi2g22810-BdFAMA   HVSSFDKLSFSDVLHFADFGPRLALNQPLSTHHPADSDNDEDSYFFRFQPSLPAAEDSDD
AT3G06120-AtMUTE      ------------------------------------------------------------
Bradi1g18400-BdMUTE   ------------------------------------------------------------


AT3G24140-AtFAMA      TQHLMPSHQTSQEGGECGGNIGNVFLEEKEDQDDDNDNNSVQLRFIGGEEEDRENKNVTK
Bradi2g22810-BdFAMA   PTAQHAAVTTQGSGGDHGTVGGGVSESTTTLVQPQQQQQETVGGGKGGGGGGAGNK----
AT3G06120-AtMUTE      ------------------------------------------------------------
Bradi1g18400-BdMUTE   ------------------------------------------------------------


AT3G24140-AtFAMA      KEVKSKRKRARTSKTSEEVESQRMTHIAVERNRRKQMNEHLRVLRSLMPGSYVQRGDQAS
Bradi2g22810-BdFAMA   ---SGRRKRPRSTKTSEEVESQRMTHIAVERNRRRQMNDYLRVLRSLMPGSYVQRGDQAS
AT3G06120-AtMUTE      -----------------------MSHIAVERNRRRQMNEHLKSLRSLTPCFYIKRGDQAS
Bradi1g18400-BdMUTE   -----------------------MSHIAVERNRRRQMNEHLKTLRSLTPALYVKRGDQAS
                                             *:*********:***::*: **** *  *::******

AT3G24140-AtFAMA      IIGGAIEFVRELEQLLQCLESQKRRRILGETGRDMTTTTTSSSSPITTVAN-------QA
Bradi2g22810-BdFAMA   IIGGAIEFIRELEQLIQCLESQKRRRLYGDAPRPTAPDISTGAGAPPVVPP-------PA
AT3G06120-AtMUTE      IIGGVIEFIKELQQLVQVLESKKRRKTLN------------RPSFPYDHQTIEPSSLGAA
Bradi1g18400-BdMUTE   IIGGAVDFIRELHVLLEALQANKRRRLNNNLHPCSTPTTPSPRSLPTNNTNSSSPGSGGS
                      ****.::*::**. *:: *:::***:  .                .            :

AT3G24140-AtFAMA      QPLIITGNVTELE-------------------------------------------GGGG
Bradi2g22810-BdFAMA   TSSMLQHEQQAAPPQGPPHHDAPAPFYVVPAPSPGTSLPLIPVISDDGVAKGIDDLDGGL
AT3G06120-AtMUTE      TTRVPFSRIE-------------------------------------------NVMTTST
Bradi1g18400-BdMUTE   SSAASNTGSG-------------------------------------------GGVNKEK
                      .

AT3G24140-AtFAMA      LREETAENKSCLADVEVKLLGFDAMIKILS-RRRPGQLIKTIAALEDLHLSILHTNITTM
Bradi2g22810-BdFAMA   GREEVAENKSCLADIEVRVLGADAVVKVLS-RRRPEQLIKTIAVLEEMHLSILHTNITTI
AT3G06120-AtMUTE      FKEVGACCNSPHANVEAKISGSNVVLRVVS-RRIVGQLVKIISVLEKLSFQVLHLNISSM
Bradi1g18400-BdMUTE   ARELAACCSSAAAEVEARISGANLLLRTLSGRAPPGQAAKMVGLLQALHLEVLHLNISTL
                      :*  *  .*  *::*.:: * : ::: :* *       *  * :. *: : :.:** **::

AT3G24140-AtFAMA      EQTVLYSFNVKITSETRFTAEDIASSIQQIFSFIHANTNISGSSNLGNIVFT--------
Bradi2g22810-BdFAMA   DQTVLYSFNVKIAGEPRFTAEDIAGAVHQILSFIDINYTL--------------------
AT3G06120-AtMUTE      EETVLYFFVVKIGLECHLSLEELTLEVQKSFVSDEVIVSTN-------------------
Bradi1g18400-BdMUTE   EDTVLHSFVLQIGLECQLSVEDLAFEVHQTFCCDYQQEDHHGQQQLLLELPIAGTVHGDI
                      ::***: * ::*  * ::: *:::  ::: :

AT3G24140-AtFAMA      ---
Bradi2g22810-BdFAMA   ---
AT3G06120-AtMUTE      ---
Bradi1g18400-BdMUTE   MIN
```

Key
bHLH domain
HER DNA binding site
FAMA SMF domain
LxCxE binding site
MUTE SMF domain

Figure S9. Protein sequence alignments of MUTE and FAMA in Brachypodium and Arabidopsis. Protein alignment of AtMUTE, AtFAMA, BdMUTE, and BdFAMA. The bHLH domain is indicated by blue text and contains the HER DNA binding domain (pink highlight). The LxCxE RBR binding site of AtFAMA, and IxCxE putative alternative site of BdFAMA are shown in yellow highlight. The ACT-like SPCH-MUTE-FAMA (SMF) structural domain is highlighted in blue; light blue for MUTE SMF and dark blue for FAMA SMF. Alignments were done using the Clustal 2.1 Multiple Sequence Alignment tool. [Supports Figure 4]

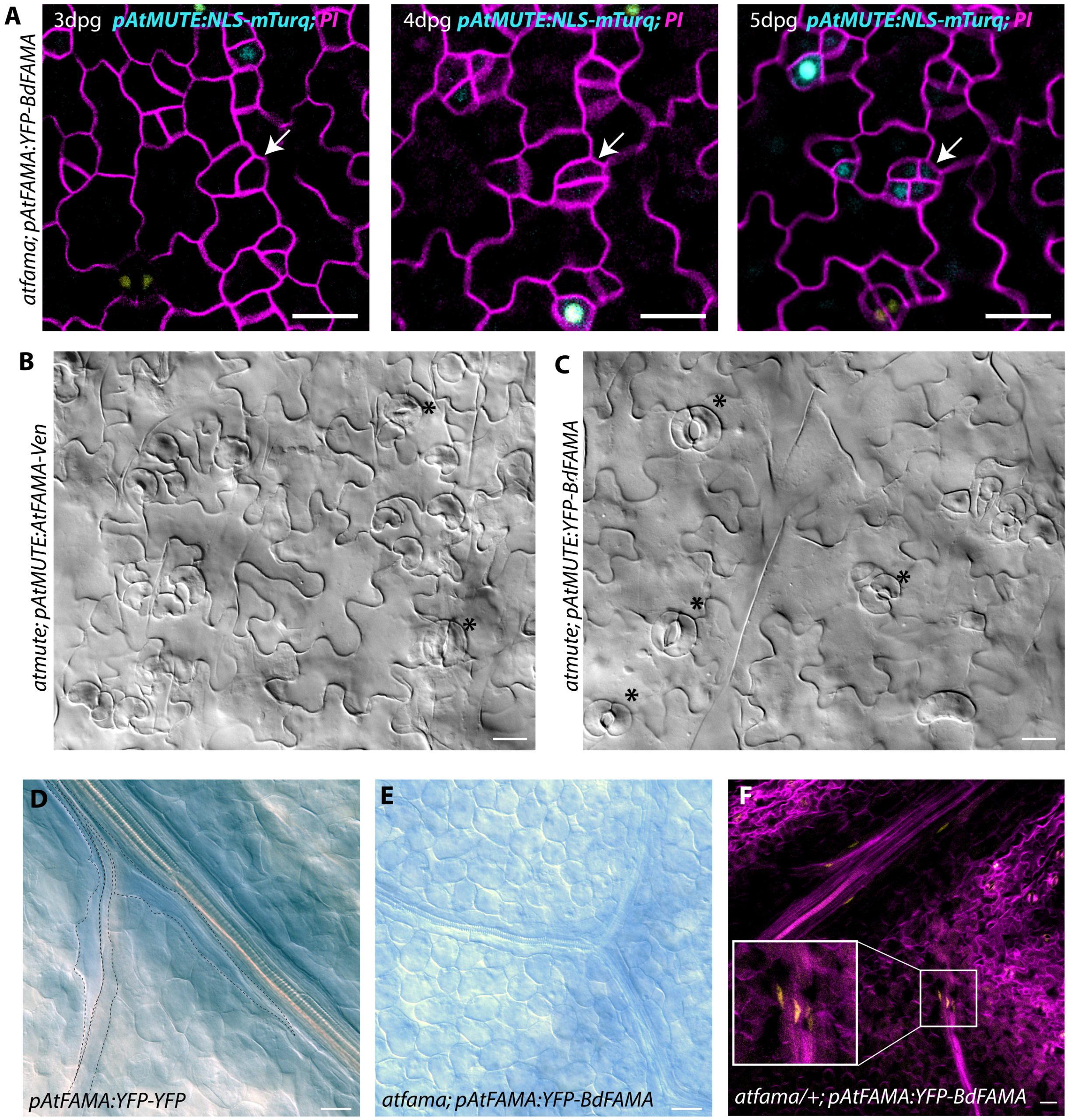

Figure S10. Additional phenotypes revealed in rescue experiments with Arabidopsis and Brachypodium stomatal bHLHs. (A) Confocal images taken in time course at 3, 4, and 5 dpg in *atfama; AtFAMA$_{pro}$:YFP-BdFAMA; AtMUTE$_{pro}$:NLS-mTurq.* White arrow points to a cell at 3 dpg that divides and expresses *AtMUTE* at low levels at 4 dpg, and divides again at 5 dpg, with all daughter cells expressing *AtMUTE.* **(B-C)** DIC images of cleared abaxial tissue from *atmute; AtMUTE$_{pro}$:AtFAMA-Ven* and *atmute; AtMUTE$_{pro}$:YFP-BdFAMA* in 10 dpg cotyledons. Black asterisks indicate stomata. **(D-E)** DIC images of tissue stained with Coomassie Brilliant Blue (CBB) to visualize the

presence or absence of myrosin idioblast (MI) cells in *AtFAMA$_{pro}$:YFP-YFP* and *atfama; AtFAMA$_{pro}$:YFP-BdFAMA*. **(D)** Developing MIs are stained blue in *AtFAMA$_{pro}$:YFP-YFP.* **(E)** *AtFAMA$_{pro}$:YFP-BdFAMA* fails to rescue the MI development defect in *atfama* and no stained MIs are observed*.* **(F)** Confocal image of abaxial tissue from true leaf *atfama/+; AtFAMA$_{pro}$:YFP-BdFAMA* stained with PI. Signal is observed in the heterozygous *atfama* background. All myrosin cell images are taken from first rosette true leaves 10–15 dpg. Scale bar = 20 µm.
[Supports Figures 5-6]

Table S1. Primer sequences used in this study.

Primer name	Sequence	Purpose	Notes
primMXA 22-FWD	GCTATGATCTCTCGCAGTCG	Genotyping BdFAMA CRISPR g4	
primMXA 24-REV	AACGGAGAGAGAGTACTACGGG	Genotyping BdFAMA CRISPR g4	
priJM_BdMUTEpro-FWD	CAGGCTAGCAGCACTATT	Genotyping BdMUTE promoter for cloning	from Raissig et al 2017
priJM_BdMUTEpro-REV	GATCGTGTCGTTCTTCct	Genotyping BdMUTE promoter for cloning	from Raissig et al 2017
priDZ44, Hyg_MRaissig_Fprim	GCGAGTACTTCTACACAGCC	Genotyping Hygromycin resistance in Brachypodium lines	from Raissig et al 2016
priDZ45, Hyg_MRaissig_Rprim	GCGAAGAATCTCGTGCTTTC	Genotyping Hygromycin resistance in Brachypodium lines	from Raissig et al 2016
priDZ46, Cas9_MRaissig_Fprim	TCGACGAACAGCTGCTTTT	Genotyping Cas9 in Brachypodium lines	from Raissig et al 2016
priDZ47, Cas9_MRaissig_Rprim	GGACAAGGGCAGGGATTTC	Genotyping Cas9 in Brachypodium lines	from Raissig et al 2016
BdSCRM2 qPCR-FWD	GCCGAGCAATGGAAGGATGGTC	qPCR primer for BdSCRM2-forward	
BdSCRM2 qPCR-REV	AGACTAAGGGCCACAGTTCCGG	qPCR primer for BdSCRM2-reverse	
BdMUTE qPCR-FWD	TCGAAGCACTCCAGGCAAACAAG	qPCR primer for BdMUTE-forward	
BdMUTE qPCR-REV	GGTGCTGCAGGGATGAAGATTG	qPCR primer for BdMUTE-reverse	
BdFAMA qPCR-FWD	TACTCCTTCAACGTCAAGATCGC	qPCR primer for BdFAMA-forward	
BdFAMA qPCR-REV	GTCGATGAAGCTAAGGATCTGGTG	qPCR primer for BdFAMA-reverse	
BdICE1 qPCR-FWD	TGGATGTCTTCAAGGCTGAGGTAG	qPCR primer for BdICE1- forward	from Raissig et al 2016
BdICE1 qPCR-REV	GACTTGGAGCAGAACTGCCTTG	qPCR primer for BdICE1- reverse	from Raissig et al 2016
priMR312	GTCACCCGCAATGACTGTAAGTTC	qPCR primer for BdUBC18 - Bradi4g00660	from Raissig et al 2016
priMR313	TTGTCTTGCGGACGTTGCTTTG	qPCR primer for BdUBC18 - Bradi4g00660	from Raissig et al 2016
priMXA3	ggcaGGCGGTCACCACCCAAGGAT	Guide 4 CRISPR Bradi2g22810 BdFAMA FP	
priMXA4	aaacATCCTTGGGTGGTGACCGCC	Guide 4 CRISPR Bradi2g22810 BdFAMA RP	
priMXA5	ATGGAAAAACAGGTCTGCTT	Cloning BdFAMA ORF	
priMXA6	CCTCCATGAGAAAGTGGAAG	Cloning BdFAMA ORF with 3' UTR	
MUTE SEQ F	ATGTCTCACATCGCTGTTGAAAGGAATCG	Genotyping *atmute*	
MUTE SEQ R	ATCGAAGCTTGATCTCCCTAATACCGATC	Genotyping *atmute*	
primMXA7	GGCGCGCCACCAGCCTAGGAGAGTTGAG	Cloning BdFAMA promoter	
primMXA8	GGCGCGCCTGATCAGAAGGAACACGTATGG	Cloning BdFAMA promoter	
gAtFAMA-fw	CACCATGGATAAAGATTACTCGGTACGTACG	cloning AtFAMA gDNA FWD	
gAtFAMA-rev	AGTAAACACAATATTTCCCAGGTTAGAGC	cloning AtFAMA gDNA REV	
fama-1 LP	TCATTCATTTGCTTCCTACGG	Genotyping atfama-1 TDNA Salk line (Salk_100073)	

fama-1 RP	CAATACAAAAAGCTCCCCTCAC	Genotyping atfama-1 TDNA Salk line (Salk_100073)	
LBb1	GCGTGGACCGCTTGCTGCAACT	Genotyping atfama-1 TDNA Salk line (Salk_100073)	
BdSPCH1_fw	CACCATGGGAGACATCGCGCTGT	Cloning of Bradi1g38650	
BdSPCH1_rev	TCACGAGAACGTTTGCTGAATCTCT	Cloning of Bradi1g38650	
BdSPCH2_fw	CACCATGGCCATGGGGGATGAC	Cloning of Bradi3g09670	
BdSPCH2_rev	TCACAAAAAGGTCTGCCGGA	Cloning of Bradi3g09670	
BdMUTE_fw	CACCATGTCGCACATCGC	Cloning of Bradi1g18400	
BdMUTE_rev	TTAATTGATCATGATGTCGC	Cloning of Bradi1g18400	
BdFAMA_fw	CACCATGGAAAAACAGTCGGAGCAG	Cloning of Bradi2g22810	
BdFAMA_rev	TCATAACGTGTAGTTGATGTCGATG	Cloning of Bradi2g22810	
BdICE1-sf1_fw	CACCATGCTGTCGGGGTTCAAC	Cloning of Bradi4g17460.1	
BdICE1-sf1_rev	CTAGATCATCGGATGGAACCC	Cloning of Bradi4g17460.1	
BdSCRM2_fw	CACCATGGAGAATTCGGTGGGGG	Cloning of Bradi2g59497	
BdSCRM2_rev	CTACATTGGGTTCTGAAGACCGG	Cloning of Bradi2g59497	
BdHLH_fw	CACCATGATGTCGAGGGAGCG	Cloning of Bradi1g63040	
BdHLH_rev	CTATATTTGCTCGTCGTCGTG	Cloning of Bradi1g63040	
BdbHLH55_fw	CACCATGGTGATGAAGATGGAGGTAGAG	Cloning of Bradi2g08080	
BdbHLH55_rev	CTACACCATGTTCATGGCACG	Cloning of Bradi2g08080	
BdbHLH112_fw	CACCATGGCACTAGTGGACGCG	Cloning of Bradi3g52150	
BdbHLH112_rev	TTACTGCGAGGCCACGAG	Cloning of Bradi3g52150	
BdbHLH145_fw	CACCATGACGCTGGATGCCGT	Cloning of Bradi5g19950	
BdbHLH145_rev	TTAACTTAAAAGTTCCGGCTGCC	Cloning of Bradi5g19950	
BdDof_fw	CACCATGATTCCCATCGATCTCCAA	Cloning of Bradi1g15420	
BdDof_rev	CCCTAGGGCAGCATGG	Cloning of Bradi1g15420	

Table S2. Summary of lines used in this study.

**Plant line**	**Plant species**	**Construct used**	**Background genotype**	**Generation imaged in paper**	**# T0 regenerants**	**# Regenerants that lived to produce seeds**
bdfama	*Brachypodium distachyon*	pEX of BdFAMA Guide 4 - sgRNA under Ubip:Cas9	Bd21-3	T3	4	3; 2 with out of frame mutation (mutant phenotype), 1 in frame (WT phenotype)
$BdFAMA_{pro}$:YFP-BdFAMA	*Brachypodium distachyon*	pEX_$BdFAMA_{pro}$:BdFAMA-YFP	Bd21-3	T3	15	13
$BdFAMA_{pro}$:3xYFPnls	*Brachypodium distachyon*	pEX_$BdFAMA_{pro}$:3xYFPnlsI	Bd21-3	T1	19	13
UBI_{pro}:BdFAMA	*Brachypodium distachyon*	pEX_Ubi_{pro}:BdFAMA ORF	Bd21-3	T0	8	2 (reporter silenced) had WT phenotype and no expression
UBI_{pro}:BdFAMA-YFP	*Brachypodium distachyon*	pEX_Ubi_{pro}:BdFAMA-YFP	Bd21-3	T0	4	1
bdmute/sid	*Brachypodium distachyon*	EMS, bdmute-1	*bdmute-1*	---	---	---
bdmute; $BdFAMA_{pro}$:YFP-BdFAMA	*Brachypodium distachyon*	pEX_$BdFAMA_{pro}$:BdFAMA-YFP	*bdmute-1*	T3	60 (only imaged ~30)	60
bdmute; $BdMUTE_{pro}$:YFP-BdFAMA	*Brachypodium distachyon*	pEX_$BdMUTE_{pro}$:YFP-BdFAMA	*bdmute-1*	T0 and T1	19	5
WT; *$BdMUTE_{pro}$:YFP-BdFAMA*	*Brachypodium distachyon*	pEX_$BdMUTE_{pro}$:YFP-BdFAMA	Bd21-3	T0 and T1	17	8
bdmute; bdfama	*Brachypodium distachyon*	pEX of BdFAMA Guide 4 - sgRNA under Ubip:Cas9	*bdmute-1*	T0	28	0
atmute/+ (G>A allele)	*Arabidopsis thaliana*	EMS	Col-0	---		
atfama/+ (Salk T-DNA line)	*Arabidopsis thaliana*	Salk T-DNA line (Salk_100073)	Col-0	---		

atfama; AtFAMA$_{pro}$:YFP-BdFAMA	*Arabidopsis thaliana*	pEX_AtFAMA$_{pro}$:YFP-BdFAMA	Col-0	T3		
atmute; AtMUTE$_{pro}$:YFP-BdFAMA	*Arabidopsis thaliana*	pEx_AtMUTE$_{pro}$:YFP-BdFAMA	*atmute*	T3		
atmute; AtMUTE$_{pro}$:AtFAMA-Ven	*Arabidopsis thaliana*	crossed with *atmute/+*	*atmute/+*	F3		
atfama; AtFAMA$_{pro}$:YFP-BdFAMA; AtFAMA$_{pro}$:NLS-mTurq	*Arabidopsis thaliana*	pEX_AtFAMA$_{pro}$:NLS-mTurq	*atfama; AtFAMA$_{pro}$:YFP-BdFAMA*	T2		
atfama; AtFAMA$_{pro}$:YFP-BdFAMA; AtMUTE$_{pro}$:NLS-mTurq	*Arabidopsis thaliana*	pEX_AtMUTE$_{pro}$:NLS-mTurq	*atfama; AtFAMA$_{pro}$:YFP-BdFAMA*	T2		
