# Supplementary material for: Expanded roles and divergent regulation of FAMA in Brachypodium and Arabidopsis stomatal development
Source: Plant Cell. 2022 Nov 28;35(2):756–75. doi: 10.1093/plcell/koac341 (PMC9940870; doi:10.1093/plcell/koac341)
Supplement: koac341_Supplementary_Data [file tpc22.00575.PeerReviewReport.pdf › tpc22.00575.PeerReviewReport.pdf]

Expanded roles and divergent regulation of FAMA in Brachypodium and Arabidopsis stomatal development

Katelyn H. McKown, M. Ximena Anleu Gil, Andrea Mair, Shou-Ling Xu, Michael T. Raissig and Dominique C. Bergmann

Corresponding author: Dominique C. Bergmann dbergmann@stanford.edu

Review timeline:

**TPC2022-RA-00575**	Submission received:	June 29, 2022
	1st Decision:	Aug. 24, 2022 *revision requested*
**TPC2022-RA-00575R1**	1st Revision received:	Sept. 27, 2022
	2nd Decision:	Oct. 26, 2022 *acceptance pending, sent to science editor*
	Final acceptance:	Nov. 22, 2022

REPORT: (The report shows the major requests for revision and author responses. Minor comments for revision and miscellaneous correspondence are not included. The original format may not be reflected in this compilation, but the reviewer comments and author responses are not edited, except to correct minor typographical or spelling errors that could be a source of ambiguity.)

TPC2022-RA-00575 1st Editorial decision – *revision requested* Aug. 24, 2022

We have received reviews of your manuscript entitled "Extracurricular roles and divergent regulation of FAMA in Brachypodium and Arabidopsis stomatal development." Thank you for submitting your best work to The Plant Cell. The editorial board agrees that the work you describe is substantive, falls within the scope of the journal, and may become acceptable for publication, pending revision and potential re-review.

We ask you to pay particular attention to the reviewers' points on BdFAMA function in a bdmute background, as all three reviewers had comments and questions about this section of the manuscript. In preparing your revision, please be aware that we are reluctant to see manuscripts undergoing multiple rounds of revision and would be unlikely to offer you more than one chance to satisfy the reviewers.

-- Reviewer comments:

TPC2022-RA-00575R1 1st Revision received Sept. 27, 2022

Reviewer comments and **author responses**:

<u>Reviewer #1:</u>

The authors describe functional diversity and similarity of the bHLH transcription factor FAMA on development of stomata in monocot and dicot grasses. By focusing on the FAMA homolog of the temperate grass *Brachypodium distachyon*, they did genetic analyses and interactome analyses. Their findings of species-specific differences are interesting beyond *A. thaliana*. I think this manuscript provides valuable information of functions of conserved bHLH transcription factors (MUTE and FAMA) in stomatal development of plants. Most of their experiments have been done adequately. However, I think this manuscript has three major difficulties, as follows.

Major points:

1. From the results with *bdmute; BdFAMApro::YFP-BdFAMA*, the authors state that *BdFAMA* is able to recruit subsidiary cells in *bdmute*. However, it is unclear whether *YFP-BdFAMA* has an ability to function in *bdmute* instead of endogenous *BdFAMA*. Complementation test with *bdfama; BdFAMApro:: YFP-BdFAMA* is necessary for the statement.

The reviewer points out an important clarification for us to make. The experiments here were done with tagged proteins and thus we should be careful to indicate that it was a YFP-tagged FAMA that could substitute for MUTE in recruiting subsidiary cells. We have modified to text appropriately. Given that *BdMUTE-/-* plants have a SC-less phenotype, we would argue that endogenous BdFAMA is not able to substitute for BdMUTE normally and the phenotype of *bdmute bdfama* double mutants compared to single mutants suggest that endogenous *BdFAMA* has some independent roles.

In terms of the complementation test recommended by the reviewer, this would seem to be to test whether BdFAMA-YFP is sufficient to rescue a *bdfama* mutant. While we appreciate that it is important to demonstrate that the GC defect is due to a mutation in BdFAMA, we think the multiple alleles reported here (and similarity to phenotypes in rice and maize published elsewhere) are reasonable evidence for this fact. In terms of whether

BdFAMA-YFP is able to rescue *bdfama* mutants, rescuing a CRISPR/Cas9 induced <u>lethal</u> Brachpodium mutant with a transgene is a challenge given the paucity of selectable markers and the timeframe for transformation and regeneration. We do show that BdFAMA-YFP is capable of rescuing both *atfama* and *atmute* mutants, and thus think it is likely that it is sufficient to rescue *bdfama*.

A second issue is whether addition of the tag might cause an artifactual rescue. YFP/GFP tags have been reported to stabilize proteins, to force dimer/polymerization, to prevent movement and to disrupt function. Considering this specific situation, when we find that BdFAMA can act to recruit subsidiary cells, we are observing it to have a role before its typical role of GC differentiation. It is possible that all that is needed to convert the function of one TF to another is stabilization, but we find this unlikely. Moreover, one of the more interesting findings, that BdFAMA does not appear in SMCs, and yet is able to induce divisions and recruit SCs required that we be able to visualize the protein.

2. *pBdFAMA::YFP-BdFAMA* is not detectable in stage 3 of the wild type [Fig. 1G (3)], while it is detectable in stage 3 of *bdmute* [Fig. 2C (3)]. This result implies that the timing of *pBdFAMA::YFP-BdFAMA* expression is earlier in the absence of BdMUTE. Quantitative analysis is necessary to clarify the point. If the timing depends on the genetic background, please explain about it.

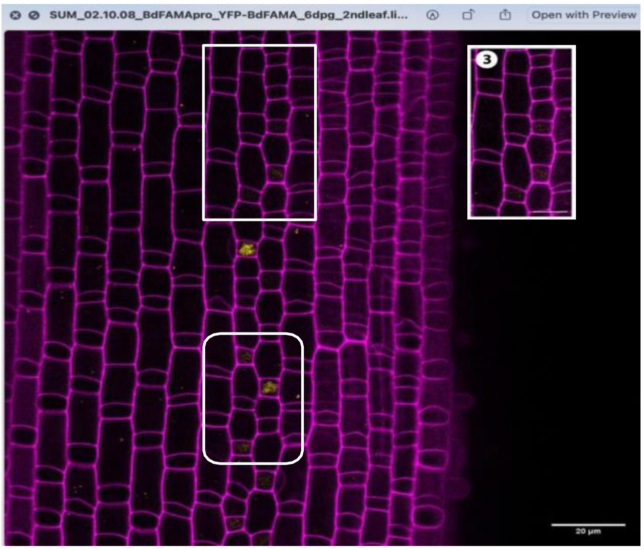

We revisited our images to see whether there was a consistent pattern to onset of expression. We found that in both WT and *bdmute* plants, BdFAMA accumulation is low and sporadic. Below is a larger field of view of a WT plant, from which the panel in Figure 1C is taken. We occasionally see a GMC with BdFAMA expression, and this sporadic nature can also be observed with BdFAMApro::3xYFP.

In the panel we included in Figure 1C, 1/5 GMCs in the box (3) are expressing BdFAMA at the slightly higher level, and the rest are dim. In this larger frame there are 2 stomatal cells that have recruited one subsidiary cell each. In the larger images you can see a couple of cells that have not yet recruited subsidiary cells that have higher

BdFAMA-YFP expression (white rounded rectangle below). Overall, the range of expression levels is comparable to what we see in *bdmute; BdFAMA$_{pro}$:YFP-BdFAMA*.

3. Please provide evidence of the naming of 'BdMUTE' and 'BdFAMA'. For example, a phylogenetic tree of bHLH Ia TF genes including *B. distachyon*, *A. thaliana*, and *O. sativa* would be helpful for the readers.

There are already several published phylogenies of these bHLH proteins, and they all concur with the placement of homologues as shown in the image below (e.g. <u>figure 3</u> or <u>Figure 1</u> or <u>Figure S3</u>). In the revision, we cited these earlier papers that confirm the BdFAMA ortholog text lines 92 and 110-12

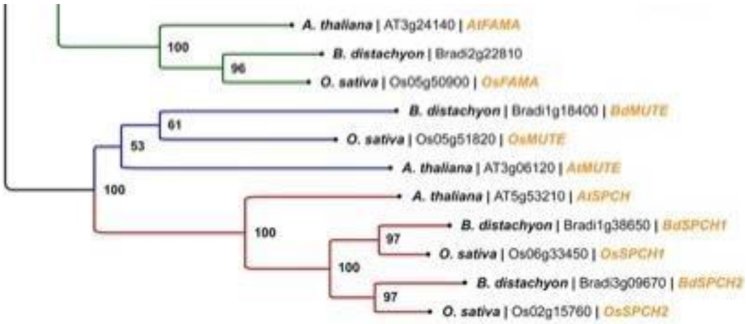

Minor points:

4. Lines 14-18: The final sentence of Abstract is somewhat difficult to read. Please make it easy for the readers to follow the statement of the authors.

   The abstract was revised and sentence structure changed for clarity.

5. Line 96: 'wildtype' should be 'wild type' throughout the text.

   Done.

6. Lines 382-383: The publication "Shirakawa et al., 2014 [PMID: 25304202]" should be cited in addition to "Li and Sack. 2014".

   Done. This certainly should have been included, and we apologize that we omitted this reference from the initial submission.

7. Lines 512-514: The authors state "We proposed that, in myrosin cells, AtFAMA is required for its own expression, as we failed ...". However, in previous publications "Li and Sack. 2014" and "Shirakawa et al., 2014 [PMID: 25304202]", it was reported that "AtFAMA is required for its own expression". Please correct the sentence by citing both publications.

   Done. Again, we apologize that we omitted this reference from the initial submission.

8. Discussion: Subtitles would be helpful for the readers.

9. Figure 1A (legend): Definitions of the stages are important in this manuscript. However, this legend has a difficulty to understand the correlations between events and stages. Please provide an explanation of each stage.

   Done

10. Figure 1 (legend): Please add a space between each number and dpg throughout the text.

    Done

11. Figure 2A: If the image corresponds to that of Fig. 1G (6), it might be better to combine panels A and B, or panel A can be deleted.

    We deleted panel A

12. Figure 2: Fig. 2D (line 163) appears before Fig. 2C (line 170) in the text. Please re-order the panels according to their appearance.

    Done (by altering text)

13. Figure 4A: The phrase "resembling the stomatal arrest state of *rice of maize mute mutants*" does not make sense. Please correct it.

    Done

14. Figure 4C: It might be better to change "*bdmute*, and *bdfama*" by "*bdfama*, and *bdmute*".

    Done

15. Figure 4: This figure is doubled. Please delete one of them.

Thank you for catching this error. It was an upload error that we have fixed.

16. Figure 5 (title): The phrase "but not is not able to fully enforce terminal fate." does not make sense. Please correct it.

Done

17. Figure 5A: 5days should be 5 days.

Done

Reviewer #2:

Extracurricular roles and divergent regulation of FAMA in Brachypodium and Arabidopsis stomatal development McKown et al.

This manuscript by McKown et al., describes the evolutionary conservation of two members of the core bHLH transcription factors regulating stomatal development. The majority of functional characterization was done in the monocot model system, Brachypodium distachyon, with important conservation comparisons to the dicot model, Arabidopsis thaliana. All of the figure images are of excellent quality and most of them clearly demonstrate the authors conclusions and model of stomatal development across species. All supplemental materials are of high quality and visually useful in understanding the conclusions of the authors. Experimental design is well-thought-out and is well-written overall.

I only have minor comments/corrections indicated below:

Minor comments/corrections

1) Fig. 4. Redundancy. I had two figure 4. One of them didn't have any significance associated with the panels.

Thank you for catching this error. It was an upload error that we have fixed.

2) The authors provide specific details (Line 163 for instance) on percentages of some cell types which seem to reflect in the representative images in the associated figure. However, in Fig. 5, the authors indicate that both AtMUTEpro::YFP-bdFAMA and AtMUTEpro::AtFAMA-Venus can rescue atmute with the conclusion that the compensatory ability of FAMA for MUTE is shared in Arabidopsis and Brachypodium (Line 374). Visually, Fig. 5D and 5E look very different, that is similarly the case in FIG S10. A plant can look visually normal (growth phenotype) while only producing two or three stomata/leaf, so although the growth phenotype is rescued the underlying stomatal differentiation has not been substantially changed.

2a) I would ask the authors to clarify/quantify the level of stomatal rescue of atmute by AtFAMA or bdFAMA. At least two of the stoma in 5E are not indicated with an asterisk, so there are at least 6 stoma and very few meristemoid cells versus 5D, which looks very much like atmute with the exception of a single stoma. I understand the growth phenotype is rescued, but how many stomata were actually produced in the atmute;AtMUTEpro:AtFAMA-venus lines? The statement that the compensatory nature of FAMA is similar between Brachypodium and Arabidopsis seems misleading given the provided data.

We appreciate the feedback on the wording of this passage and acknowledge that the micrograph images contribute to the confusion. We have substituted the image in Figure 5 for one that better illustrates a typical field. For the purposes of our question, we wanted to know whether AtFAMA and BdFAMA were capable of generating morphologically normal stomata when supplied in place of AtMUTE, as previous reports had suggested that they could not. The qualitative data we show are sufficient to demonstrate this.

We observed numerous stomata in both rescue lines, although BdFAMA produced more 2-celled stomata and AtFAMA produced more single guard cells, and development of the stomatal lineage was slightly delayed in the latter case. We do not feel comfortable making any quantitative claims about the degree of rescue because we cannot ensure that BdFAMA and AtFAMA are expressed at precisely the same levels, nor can we tell if protein stability plays into the degree of rescue. We did notice that AtFAMA tended to produce more single guard cells,

which could indicate a more traditional "FAMA" role compared to the 2-celled stomata produced by BdFAMA (an activity more often ascribed to "MUTE").

3) There is a typo in the title of Fig. 5.

Fixed

4) I found the word "Extracurricular roles" in the title to be an odd word choice to indicate a shift of the current model of FAMA function from the last ~15 years of research. "nonestablished roles" or "additional roles". I feel the use of Extracurricular in the title will falsely attract those looking for biology education articles.

We have changed this to "expanded"

Reviewer #3:

This is a very interesting manuscript that gives insight into differences in the roles of FAMA and MUTE in grasses (Brachypodium) vs. dicots (Arabidopsis), as well as revealing previously unnoticed overlap in the roles of the two genes in Arabidopsis. The authors also show that AtFAMA has gained a new role in regulating development of a Brassicaceae-specific cell type that BdFAMA cannot substitute for. This work has important implications not only for understanding a cell differentiation pathway that is essential for all land plants, but also for understanding how that pathway has diverged in the grasses, which have their own specialized pathway of stomatal development.

Finally this work is also important for understanding the acquisition of novel functions by transcription factors in evolution. Thus this work is highly significant and should be of broad interest.

In general, the quality of the work is excellent and supports the conclusions, and the manuscript is well-written. I have just a few questions.

1. Given that BdFAMA can only partially substitute for AtFAMA in atfama- mutants, rescuing GC differentiation but failing to completely block division and establish terminal differentation, I am left wondering about mechanism. The work as it stands is quite nice, and I am not sure how much additional work it is fair to request but two obvious testable mechanisms suggest themselves. One possibility is that BdFAMA does not bind to RBR, or binds only weakly. Alternatively, the two FAMAs have slightly different DNA binding preferences. This group has used Y2H and BiFC to show that AtFAMA binds to RBR, so it should be fairly easy and quick to test whether BdFAMA and AtRBR interact in these assays. One could also express a BdFAMA mutated to introduce the LXCXE sequence, and ask whether this is sufficient to fully replace AtFAMA function. Testing binding site specificity would be more work- DAPseq using the two transcription factors on At genomic DNA comes to mind. Again, I am not sure how much work is fair to request, but these questions of mechanism seemed obvious to me, and could at least be commented on.

   These are excellent points and we have debated about these experiments for the last two years. Unfortunately, bHLHs (particularly heterodimeric ones) do not work well in the DAP-seq assays (Bartlett et al., 2017).

   We revisited our experiments where we mutated the AtFAMA LxCxE motif (Matos et al., Elife) to assess how much sensitivity the various in vivo and in vitro assays have to determine differential binding of the LxCxE and IxCxE motifs. We found that expression variability in BiFC experiments and the overexpression intrinsic to Y2H are too great to see the subtle changes we might expect for this substitution.

2. I found the section starting on line 283 on regulation of FAMA by MUTE a bit unclear, particularly as regards to the differences between Brachypodium as compared to maize and rice. In lines 285-288, the authors state that in maize and rice, "FAMA expression is reduced significantly in mute backgrounds...our observations ...suggest that there may be a different regulatory strategy in Brachypodium." This sentence reads as if MUTE does not have a significant role in regulating BdFAMA, but in fact BdFAMA is significantly reduced in bdmute compared to WT. On looking up the maize and rice papers, I see that in those plants, FAMA expression is almost totally dependent on MUTE, while there is substantial BdFAMA expression remaining in the mute mutant. And some of this is clarified by the end of the paragraph discussing the results. Nevertheless, I was initially confused when I jumped ahead to the data, and saw dependence on MUTE of FAMA expression. It could have been made clear from the outset that the

dependence was near total in the other grasses, and this would have helped me. I suggest some rewriting of this paragraph.

We rewrote this section to clarify the relationship between BdMUTE and BdFAMA expression relative to the case of their homologues in other plants in lines 350-363.

3. I think something is wrong with the Ohashi-Ito reference in the reference list. When I looked up the paper to check something, I see that on the Plant Cell web site, it only lists Ohashi-Ito and Bergmann as authors, but in the reference list, several other authors are listed, and Bergmann is listed twice.

We rechecked our reference list to fix this (and other) errors.

TPC2022-RA-00575R1 2nd Editorial decision – *acceptance pending* **Oct. 26, 2022**

We are pleased to inform you that your paper entitled "Expanded roles and divergent regulation of FAMA in Brachypodium and Arabidopsis stomatal development" has been accepted for publication in The Plant Cell, pending a final editorial review by a science editor.

Final acceptance from Science Editor **Nov. 22, 2022**
